# Three Parts of the Plant Genome: On the Way to Success in the Production of Recombinant Proteins

**DOI:** 10.3390/plants12010038

**Published:** 2022-12-21

**Authors:** Sergey M. Rozov, Alla A. Zagorskaya, Yuri M. Konstantinov, Elena V. Deineko

**Affiliations:** 1 Federal Research Center, Institute of Cytology and Genetics, Siberian Branch of Russian Academy of Sciences, pr. Akad. Lavrentieva 10, Novosibirsk 630090, Russia; 2 Siberian Institute of Plant Physiology and Biochemistry, Siberian Branch of Russian Academy of Sciences, Lermontova Str. 132, Irkutsk 664033, Russia

**Keywords:** transgene plants, transplastomic plants, mitochondrial genome, recombinant proteins

## Abstract

Recombinant proteins are the most important product of current industrial biotechnology. They are indispensable in medicine (for diagnostics and treatment), food and chemical industries, and research. Plant cells combine advantages of the eukaryotic protein production system with simplicity and efficacy of the bacterial one. The use of plants for the production of recombinant proteins is an economically important and promising area that has emerged as an alternative to traditional approaches. This review discusses advantages of plant systems for the expression of recombinant proteins using nuclear, plastid, and mitochondrial genomes. Possibilities, problems, and prospects of modifications of the three parts of the genome in light of obtaining producer plants are examined. Examples of successful use of the nuclear expression platform for production of various biopharmaceuticals, veterinary drugs, and technologically important proteins are described, as are examples of a high yield of recombinant proteins upon modification of the chloroplast genome. Potential utility of plant mitochondria as an expression system for the production of recombinant proteins and its advantages over the nucleus and chloroplasts are substantiated. Although these opportunities have not yet been exploited, potential utility of plant mitochondria as an expression system for the production of recombinant proteins and its advantages over the nucleus and chloroplasts are substantiated.

## 1. Introduction

Plants have accompanied humans throughout their historical development, and their role in human life can hardly be overestimated. Plants are not only “the lungs” of our planet and the most important source of food, energy, and raw materials in everyday life but also the source of manifold valuable therapeutic compounds traditionally used by humankind for the management of various maladies and diseases. The current relevance of plants is additionally emphasized by their utility as alternative expression systems for bulk production of recombinant proteins, including those for medical purposes. Thus, we cannot but admit that the resolution of such global challenges as the food, energy, ecological, and drug security is unfeasible without broad application of plants in the already existing areas of biotechnology as producers of a long list of biologically active compounds.

Currently, a large number of pharmaceutically valuable proteins are synthesized as recombinant analogs in different expression systems (*Escherichia coli*, yeasts, and animal cells) rather than extracted from natural sources. This makes it possible to manufacture on a commercial scale the proteins unobtainable by traditional extraction methods (for example, insulin or human growth hormone). It should be underscored that approximately 80% of recombinant pharmaceutically valuable proteins are synthesized in mammalian cell cultures [1]. Although this expression system is prevalent in the manufacture of recombinant proteins, it has its own shortcomings associated with the risk of contamination with pathogens, expensiveness, formation of side products, and other problems.

Plant expression systems are the most attractive for researchers and pharmaceutical companies. Advantages of plant expression systems consist of relatively inexpensive cultivation; the absence of undesirable components, such as bacterial endotoxins or hyperglycosylated target proteins, produced by yeasts; and, in contrast to animal cell cultures, the absence of animal and human pathogens. Plant cells enable correct folding and formation of intricate multimeric protein complexes as well as most of the post-translational modifications of target proteins necessary for their biological activity [2]. Plant cells are able to synthesize complex mammalian proteins, such as collagens, hemoglobin, and immunoglobulins. Numerous methods and technologies of recombinant protein production in plant expression systems involving various plant species have been designed so far, in addition to the techniques for gene transfer based on stable or transient expression systems. In general, this research direction is referred to as molecular farming, whereas the modified plants synthesizing recombinant proteins are known as molecular biofactories.

Molecular farming as a promising alternative approach to recombinant protein production is currently one of the most rapidly developing segments of the world economy. The largest biotechnological and pharmaceutical companies, such as Protalix Biotherapeutics (Israel), Synthon (The Netherlands), Ventria (United States), Medicago (Canada), Greenovation (Germany), and Pfizer (United States), take a keen interest and invest considerable amounts of money in the research and development of new platforms for the manufacturing of valuable recombinant proteins. As of today, approximately 50 new medical treatments based on recombinant proteins (produced in plant systems) have been developed and tested, and over 35 new technologies utilizing plant expression systems have been patented by drug designers [3].

The molecular biofactories based on the technologies of recombinant DNA require the transfer of target genes into the plant genome and the use of its transcriptional and translational machineries for biosynthesis of a recombinant protein. Note that biosynthetic potential of a plant cell is determined by the operation of three molecular machineries, namely, nuclear, plastid, and mitochondrial ones. When transgenic plants are constructed, a target gene coding for an important recombinant protein can be integrated into the nuclear genome (providing stable expression) or used as a template for temporary synthesis of the target product (transient expression). If a target gene is delivered to and integrated into the chloroplast genome, the plants of this type are referred to as transplastomic plants. Although transgenic and transplastomic plants have so far been used as biofactories, the potential of the transcriptional and translational machinery of mitochondria is arousing increasing interest in researchers.

In contrast to other living organisms, the genome of plant cells comprises three parts, represented by the genomes of the nucleus, chloroplasts, and mitochondria, which have formed during evolution via the capture of proteobacteria and cyanobacteria by ancestors of eukaryotes and their subsequent adaptation as endosymbionts. This adaptation was accompanied by a reduction in endosymbiotic genomes, that is, the transfer of some of their genes to the host genome. Although the plastid and mitochondrial genomes are much smaller as compared with the nuclear genome, they still have their own systems for transcription and translation. Nevertheless, most of the endosymbiotic proteins, including the proteins of the transcriptional and translational machineries, are synthesized in the cytoplasm and only then are transported to plastids and mitochondria.

The goal of this review was to consider the potential of three molecular machineries of the plant cell for the production of various recombinant proteins whose use may replace natural sources of the corresponding proteins. Because plants have served as biofactories for over several decades, the main emphasis in this review is on the assessment of the biosynthetic potential of each of the three molecular machineries, with a focus on documented accomplishments and estimation of their biosynthetic prospects for the accumulation of recombinant proteins. In this paper, we attempt to evaluate the achieved productivity in terms of recombinant proteins in plant expression systems in order to outline the range of yet unsolved problems and to examine feasible ways to resolve them.

## 2. Specific Features of the Organization of Nuclear–Cytoplasmic Transcriptional and Translational Machinery of the Plant Cell

### 2.1. Organization of Nuclear-Cytoplasmic Molecular Machinery for Protein Biosynthesis

The molecular machineries of all organisms involved in protein biosynthesis, both in prokaryotes and eukaryotes, function based on transcription and translation. An important specific feature of the organization of the eukaryotic molecular machinery consists of the spatial separation of transcription and translation: the templates synthesized in the nucleus (mRNAs) are conveyed from the nucleus through nuclear pores to the cytoplasm, which houses protein biosynthesis and their subsequent folding and post-translational modifications. In general, the molecular machinery underlying the protein biosynthesis in the plant cell is the most intricate molecular mechanism, comprising several separate but tightly interacting entities. This machinery is composed of many molecular systems, such as the DNA replication and repair system, the transcription and translation system, the proteasome and ubiquitin signaling system (involved in regulated proteolysis), and exosomes (regulating RNA degradation). The nuclear pore complex (implementing nuclear–cytoplasmic transport of the key informational biopolymers) and the systems of cell membranes (including the endoplasmic reticulum) are the major players in the normal operation of the transcriptional and translational machinery. The RNA interference system provides transcription and translation regulation at the level of individual genes and chromatin remodeling.

In contrast to prokaryotes, whose genome has an operons-based gene organization and compact mutual arrangement of genes, the characteristic features of the eukaryotic genome are monocistronic gene structure and nonuniform distribution of genes across the genome. For instance, 90% of all wheat genes are situated in 30% of the genome space and 40% of all rice genes are concentrated in 25% of rice genome [4]. Most genes are arranged in rather short clusters, alternating with regions of repeated DNA sequences. Note that the density of gene distribution may differ between chromosome regions. For example, approximately 60% of wheat genes are localized to distal chromosome regions. The proportion of the plant genome housing genes decreases with an increase in its size, whereas the proportion of intergenic regions alternating with genes goes up correspondingly [4]. The characteristic property of some protein-coding genes is a split structure (exons and introns). Thus, the synthesis of a protein molecule from such a split template requires additional molecular machinery that would excise the introns and restore its integrity. In the eukaryotic cell, this machinery is represented by spliceosomes; they not only guarantee the integrity of the template conveyed to ribosomes but also ensure its variation via alternative splicing. This phenomenon considerably expands the structural and functional diversity of synthesized proteins. Therefore, the teamwork of numerous associated molecular processes is responsible for protein biosynthesis out of the nuclear genome.

### 2.2. Nuclear–Cytoplasmic Transcriptional and Translational Machinery as the Tool for the Biosynthesis of Recombinant Proteins

An insight into specific features of the operation of nuclear–cytoplasmic transcriptional and translational machinery is necessary not only to understand its importance for the life of the cell but also in terms of its application as a tool for the biosynthesis of recombinant proteins.

The technology for the production of recombinant proteins in plant cells is currently being developed in two directions: the use of whole plants with the delivery of recombinant genes coding for target proteins to the nuclear genome for stable or transient expression and the cultivation of genetically modified plant cells in closed systems of bioreactors. During the last 10 years, the use of cell-free systems for the biosynthesis of recombinant proteins has attracted the attention of researchers; these systems are merely components of transcriptional and translational machinery functioning outside the cell. The components of cell-free protein synthesis are recombinant genes encoding a target protein and the cell lysate containing the transcriptional and translational machinery along with the necessary substrates and ATP regeneration components providing the function of this cell-free system. Let us separately consider the capabilities of each of the above-mentioned expression systems for producing recombinant proteins in terms of efficiency of the nuclear–cytoplasmic transcriptional and translational machinery.

#### 2.2.1. Stable Expression of Recombinant Proteins in Transgenic Plants

During the last 30 years, a successful use of the stable expression of recombinant genes integrated into the nuclear genome of plant cells has been convincingly demonstrated by means of examples of improved agronomic traits of agriculturally important plant species. The created genetic constructs have included both individual genes and a set of genes the expression of which refines the characteristics of the produced transgenic plant, potentially forming the basis for the application of such plants as biofactories for the production of recombinant proteins. The examples of construction of the transgenic plants’ resistant to herbicides, different diseases, and insects, as well as tolerance to drought and salinization, are well-known and described in several reviews [5,6,7]. Stable expression of transgenes integrated into the nuclear genome has allowed to obtain plants with improved nutrient characteristics, including “golden rice” and “golden bananas”, with an increased level of β-carotene (precursor of vitamin A) [8,9,10]; tomatoes containing squalene, phytosterols, α-tocopherol, and carotenoids in their fruits [11]; and transgenic rice and sorghum with elevated contents of zinc, iron, and folic acid [12]. Despite certain contradictions in the public attitude toward genetic modification of plant genomes and strict systems of relevant regulations adopted in many countries, these technologies have manifested high commercial potential, not fully employed so far.

The field growth of transgenic plants featuring stable expression of a protein using available agrotechnical schemes offers almost unlimited opportunities for scaling up (along with minimum costs) and are optimally suitable for manufacturing products having a large existing market, e.g., enzymes and hormones (for example, gastric lipase and insulin), as well as antibodies and vaccines for disease prevention, especially in developing countries with large populations [13]. Transgenic plants are appealing for the creation of oral vaccines because antigens can be synthesized in edible tissues [14].

Considering the overall potential of molecular farming for the manufacture of recombinant proteins, it is necessary to distinguish two avenues of research in the development of this technology established so far. On the one hand, transgenic plants carrying the genes that code for therapeutic proteins are of interest to the pharmaceutical industry as a source of raw materials to extract target recombinant proteins. In particular, strengthening of the provisions requiring a decrease in the use of antibiotics in fish, meat, and dairy farming draws attention to transgenic plants as a source of recombinant vaccines [15,16]. Several reviews [17,18,19,20] have briefed the potential of transgenic plants with stable expression of target genes for manufacturing recombinant pharmaceutically important proteins. Table 1 lists some pharmaceutical proteins produced in this way along with data on their accumulation efficiency in plant tissues.

On the other hand, transgenic plants can also be utilized as a source of nonpharmaceutical proteins, such as technical enzymes and research reagents, as well as cosmetic products [18,37,38]. Molecular farming is most intriguing for the production of nonpharmaceutical recombinant proteins because of its lower costs (less market promotion of such a commercial product is needed). Using transgenic maize plants carrying grains accumulating recombinant avidin, ProdiGene, Inc. (College Station, TX, USA) has concluded after some estimates that it is feasible to produce this protein in plants as compared with the routine protocol (chicken eggs), taking into account the costs of plant growth and the isolation and purification of the recombinant protein from plant extracts [39].

The range of applications of recombinant nonpharmaceutical proteins synthesized in transgenic plant tissues is rather wide. Recombinant trypsin of a plant origin is in demand both in the food industry and in the manufacture of consumer goods (e.g., animal skin tanning), as well as for the separation of subsidiary proteins and peptides when recombinant proteins are purified. Recombinant trypsin is also used in proteome analysis to cleave proteins into peptides. State-of-the-art production of recombinant trypsin involves the biosynthesis of inactive trypsinogen in maize seeds using the globulin-1 gene promoter and an optimized signal sequence of the barley α-amylase gene as the regulatory element; this approach has allowed to increase the yield of the target protein to 3.3% of total soluble protein (TSP) [40]. Examples of the use of the recombinant proteins of an animal origin that are synthesized in plant cells are well-known, e.g., collagen, keratin, silk, and elastin, which possess high strength, rigidity, elasticity, and biocompatibility, indicating their utility for manufacturing novel stable biopolymers [41]. Transgenic maize plants with a recombinant enzyme called phytase (cleaving phytates, thereby improving the digestibility of plant fodder) have been commercialized and certified as biologically safe [42]. Table 2 presents some commercialized technologies for the manufacture of recombinant nonpharmaceutical proteins on a plant platform, including enzymes for the production of biofuel, paper, various research reagents, cosmetics, and other substances.

#### 2.2.2. Stable Expression of Recombinant Proteins in Cultured Plant Cells (in Bioreactors)

Genetic constructs carrying genes of target proteins are successfully employed as templates for the production of recombinant proteins based on stable operation of nuclear–cytoplasmic transcriptional and translational machinery of the genetically modified plant cells cultivated in closed systems of bioreactors. As compared with the open-field cultivation of transgenic plants, the culturing of plant cells in a bioreactor completely frees the production of recombinant proteins from the problems associated with changes in weather, soil, pests, and the drift of transgenes into the environment [55]. The short cycles of cell growth in suspension culture reduce the period necessary for the production of recombinant proteins to several weeks versus several months necessary for their production in transgenic plants. In addition, the cultivation of plant cells in sterile and controlled media, such as a bioreactor system, enables fine-tuning of the cell growth conditions and quality of the product in different batches. Another advantage of the protein manufacture in plant cell cultures is that recombinant proteins can be secreted from cells into the culture medium, making their subsequent extraction and purification considerably less expensive as compared with whole plants. That is why the production in bioreactors is currently regarded as one of the most promising and attractive systems for manufacturing recombinant proteins.

Over the last 10 years, the biofarming of pharmaceutical drugs in plant systems has achieved considerable success [56,57,58]. The main projects in the area of commercial production of pharmaceutically important recombinant proteins on the platform of higher-plant cell cultures are based on cell lines of tobacco (*Nicotiana tabacum* L.) BY-2 (cv. Bright Yellow-2) [59,60], rice (*Oryza sativa* L.), and carrot (*Daucus carota* L.) [61,62] (Table 3).

As evident from Table 3, the performance of the cell cultures used for commercial production of recombinant proteins amounts to 18.0–43.7 mg/L. Cell cultures of these plant species grow rapidly and are rather amenable to agrobacterial transformation. The tobacco BY-2 line has made it possible to construct (using CRISPR/Cas9 genome editing and glycoengineering) a line devoid of protein glycosylation of the plant type: plant-specific β(1,2)-xylose and α(1,3)-fucose residues [59,60]. Nonetheless, the initial BY-2 line remains in demand for the production of recombinant proteins [61].

Cultured rice cells are utilized for the production of manifold recombinant proteins, including growth factors, enzymes, interferons, and antibodies [62,63,64,65,66,67,69]. A sugar-inducible promoter system has been designed for rice cell culture; this system separates in time the phases of culture growth and product accumulation, thereby raising the yield of a recombinant protein [68]. The first commercially available protein product, taliglucerase alfa (Elelyso^®^), has been synthesized in carrot cell culture [70]. Protalix Biotherapies has designed a new type of single-use bioreactor for large-scale manufacture of this product. The history of its development and of enhancement of the platform for manufacturing recombinant proteins based on carrot cell cultures is detailed in a review by Rosales-Mendoza et al. [71].

Cell cultures of other plant species are also of interest for the production of recombinant proteins [72]. In particular, a distinctive characteristic of cultures of barrel clover (*Medicago truncatula* Gaertn.) mature cotyledons, leaves, or roots is a considerably lower content of proteases in the culture medium as compared with the tobacco BY-2 cell line [73]. This is intriguing in terms of an elevated recombinant protein yield.

Although cultured plant cells are in demand for manufacturing pharmaceutically important proteins, problems with improving the yield of recombinant proteins remain relevant for the systems based on stable expression. Of special interest are approaches that involve a combination of stable and transient expression, making it possible not only to increase the yield [74] but also to refine its quality [75]. The latter is achieved by the cocultivation of transformed plant cells whose genome contains an integrated target gene and *Agrobacterium tumefaciens* carrying additional elements that optimize its expression and post-translational modifications.

#### 2.2.3. Transient Expression

The attractiveness of (and widespread demand for) transient expression systems can be explained by (1) relative simplicity of the delivery of the template coding for a recombinant protein to plant tissues; (2) a high yield of the recombinant protein; and (3) high scalability of this system. The last feature is especially relevant for rapid production of recombinant proteins. Genetic constructs are delivered to the intercellular space of leaf tissues (this space accounts for up to one-third of their volume) by vacuum-assisted agroinfiltration [76]. This mode applied to the delivery of agrobacteria offers better access to plant cells and higher efficiency of transfer of target genes (via T-DNA) to the nucleus at the expense of elevated plant cell proliferation [77]. The increase in plant cell proliferation and the corresponding delivery of a large number of templates of the target gene to the plant cell genome are also achievable via introduction of some signal sequences of plant viruses into genetic constructs [76,78]. Once introduced, these sequences not only enhance the spread of the delivered template as T-DNA among cells but also protect it from a post-translational inactivation [79,80]. Tyurin et al. [78] have reviewed specific features of the design of genetic constructs aimed at delivery of target genes for transient expression. The technologies for the manufacture of recombinant proteins via transient expression of a target gene have been devised based on the agroinfiltration protocol [81] and adapted to two plant species, *N. benthamiana* and *N. tabacum*, by several companies for rapid vaccine production. Note that this technology has so far been adapted to 40 plant species, including trees [78].

D’Aoust et al. [82] were first to confirm that the transient expression system for recombinant proteins holds promise when a rapid response and production of vaccines are necessary to protect the population from disease outbreaks. Large amounts of antigens (50 mg/kg) of influenza virus H5N1 (avian influenza virus) and H1N1 (swine influenza virus) were produced in *N. benthamiana* plants <3 weeks after the virus sequence was isolated. These influenza vaccines have successfully passed phase II clinical trials. Fraunhofer USA Inc. has also used transient transformation (i.e., transfection) of plants for rapid production of considerable amounts of a recombinant protein by means of an isolated sequence of influenza virus H1HA [83,84]. The first experimental “cocktail” against the Ebola virus (a mixture of three monoclonal antibodies synthesized in tobacco plants via transient expression), named ZMapp^TM^, has been approved by the Food and Drug Administration (FDA) for relevant emergency situations [85]. Medicago (Québec, Canada/Durham, NC, USA) and iBio/Caliber Therapeutics (Bryan, TX, USA) in collaboration with Kentucky Bioprocessing (Owensboro, KY, USA) have designed large-scale automated plants for manufacturing recombinant products on a transient expression platform.

As an ultimate leader in the vaccine design based on transient expression, Medicago has created a unique technology for a rapid response to global health emergencies. To enhance the efficiency and immunogenicity of newly developed recombinant vaccines, this company has synthesized virus-like nanoparticles composed of immunogenic recombinant proteins [86]. At present, Medicago is developing a vaccine candidate against rotavirus infections, conducting preclinical trials of a vaccine against a highly contagious norovirus causing outbreaks of gastroenteritis [87], and completing phase II and III clinical trials of the vaccines against seasonal influenza virus strains [88].

A case study of the COVID-19 pandemic has clearly shown that emergencies very rapidly deplete production capacity for immunogenic proteins because the manufacture of other drugs and diagnostic tools cannot be stopped or delayed. These are specific situations when a transient expression is in high demand for rapid production of the necessary recombinant proteins. The expression systems of this kind help to organize the production by combining processes of preparing plants for agroinfiltration, identifying the genome of the corresponding pathogen, and isolating the antigen sequence to be multiplied in the cells of the prepared plants. This notion has given rise to the Pharma-Factory Project, funded by the European Commission and aimed at ensuring rapid advances in the area of transient expression in plants, including new expression vectors, *Agrobacterium* strains, and plant cultivars, as well as the corresponding protocols (https://pharmafactory.org accessed on 9 November 2022) [89,90]. The consolidated efforts of researchers aimed at designing vaccine candidates against SARS-CoV-2 over a short period are unprecedented [91,92,93,94]. As early as several months into the pandemic, Medicago reported the production of virus-like nanoparticles based on the S protein of SARS-CoV-2 in *N. benthamiana* plants. Analogous work has been conducted using S, E, and M proteins as immunogens, some of which have been fused to the receptor-binding domain (RBD) and expressed in *N. benthamiana* [95,96]. A transient expression system is currently used to design vaccine candidates against hepatitis B and C [97,98] and the most hazardous human papillomavirus strains [99], as well as to create a wide range of neutralizing antibodies against human immunodeficiency virus [100].

The transient expression system is even more convenient for the manufacture of recombinant proteins because of the coexpression of several genes of complex proteins, such as specific anticancer monoclonal antibodies [101]. Expression systems of this kind are regarded as promising for the manufacture of low-molecular-weight toxins, such as viscumin and ricin, used in conjugates with antibodies [102,103] and may be an ideal platform for the biosynthesis of immunotoxins [104].

#### 2.2.4. Cell-Free Expression Systems for Recombinant Proteins

**Figure 1:**
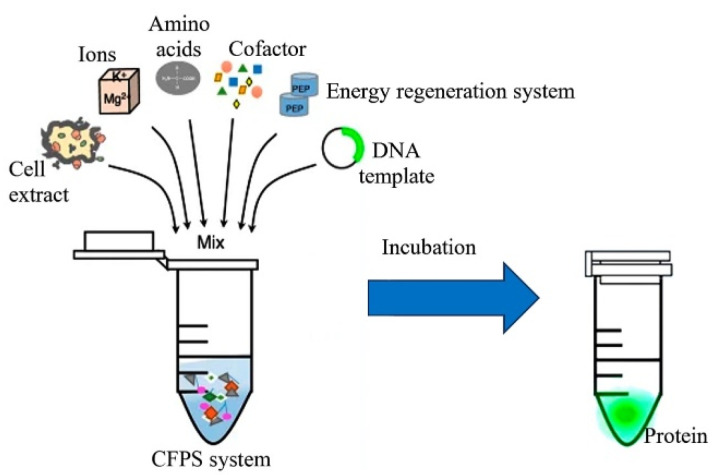
The cell-free protein synthesis (SFPS) system.

As early as 1961, Nirenberg and Matthaei [105] demonstrated that the translational machinery isolated from the cell can perform its function (and were awarded the Nobel Prize for Medicine and Physiology). In their study, the addition of an exogenous mRNA template coding for a target protein to the ribosomes and other auxiliary elements extracted from *E. coli* triggered the operation of the translational machinery and initiated the synthesis of the recombinant protein. Although the yield of the proteins synthesized according to their initial protocol was very low (as a rule, in the range of several tens of micrograms per milliliter), our understanding of the factors that limit the performance of cell-free systems for protein synthesis and how to counteract them has advanced considerably since then. Combined optimization of reaction conditions of template preparation techniques, and of ATP regeneration schemes, has laid the foundation for cell-free protein synthesis systems with an efficacy of several milligrams of a recombinant protein per milliliter within several hours. The main components of the reaction mixtures for cell-free protein synthesis comprise the following four groups: the template DNA encoding a target protein; a cell lysate containing the translational machinery and auxiliary factors; substrates for transcription and translation, including nucleotides and amino acids; and components necessary for ATP regeneration (Figure 1). Nonetheless, the functioning and replication of all these components are maintained in the cell in an automated manner; accordingly, in a cell-free system, it is necessary to somehow ensure their maintenance and uninterrupted operation. Therefore, before this technology can be accepted as an alternative platform for recombinant protein production, several problems have to be resolved: quality control of the components of the translation machinery and high costs of the reagents. The main difficulty encountered when cell-free systems are designed for protein synthesis is the high sensitivity of linear DNA to endonucleases. Numerous solutions to this problem have been investigated, namely, elimination of genes coding for endonucleases from the genome of the cells used for preparing the cell extract, mRNA stabilization, and a decrease in the reaction temperature of the cell-free synthesis in order to lower the activity of residual RNases in the extract [106].

Several cell-free systems involving lysates of rabbit reticulocytes, Chinese hamster ovary cells, HeLa cells, yeast cell extracts, or wheat embryonic cells have been proposed by investigators [107]. A cell-free expression system, BYL, based on tobacco BY-2 cells [108] looks promising for this purpose; this platform represents mechanically destroyed protoplasts with vacuoles removed beforehand (they contain nucleases and proteases diminishing the yield of the synthesized recombinant protein) [109]. The BYL system contains actively migrating microsomes formed as a result of destruction of the endoplasmic reticulum during preparation of the lysate. Protein products with incorporated N-terminal peptides can be targeted to microsomal vesicles, thereby ensuring the formation of disulfide bonds and effective folding and assembly of complicated and multimeric proteins, such as enzymes, full-fledged antibodies, and even membrane proteins. In addition, this system supports glycosylation.

LenioBio GmbH (Düsseldorf, Germany) has commercialized the BYL system (trademark ALiCE^®^) [110]. On this platform, recombinant proteins are synthesized in a reaction mixture of 1 to 10 L with a yield of tens of grams of a target protein within one batch. The lysates of tobacco BY-2 cells are able to in vitro synthesize recombinant proteins with a yield of up to 3 mg/mL, which is approximately 15-fold higher as compared with other eukaryotic cell-free systems [107] and is especially promising for the production of recombinant proteins potentially toxic to plant cells [111,112].

### 2.3. Assessment of Biosynthetic Potential of Nuclear-Cytoplasmic Transcriptional and Translational Machinery: Problems and Possible Solutions

Although successes of the development and improvement of plant expression platforms for synthesizing recombinant proteins are quite impressive, the proportion of such proteins among manufactured commercial products is still small as compared with the other expression systems. The main hindrance to widespread adoption of molecular farming technologies is their poor performance: a protein yield of 0.01 to 100 mg/L [113,114].

The problems with increasing the recombinant protein production in plant cells have so far been solved via optimization of components and processes in the following areas: (1) refining the genetic constructs that carry a target gene; (2) optimization of the performance of nuclear–cytoplasmic transcriptional and translational machinery; and (3) improvement of cost efficiency at all stages of recombinant protein production in the plant expression system. Artificially created templates that may be optimal for the functioning of nuclear–cytoplasmic transcriptional and translational machinery are upgraded by the classic methods of codon frequency optimization with regard to the system used for expression [115,116]. The yield of a recombinant protein can be increased by means of strong regulatory elements (promoters and enhancers) introduced into a genetic construct, as well as through the addition of introns into the coding region of a target gene [117].

The specific features of the organization of the plant nuclear genome and a random distribution of insertions across the genome can reduce the biosynthetic potential of an optimized genetic construct if it is inserted into regions with low transcriptional activity or regions in which exogenous DNA is recognized as foreign and inactivated. Thus, the efforts of researchers aimed at the optimization of a recombinant protein yield via the optimization of the DNA template may well be futile in the regions with suboptimal performance of the transcriptional machinery. Accordingly, this particular stage of research focused on the search for genome regions with high transcriptional activity is crucial when scientists create plant lines for the production of recombinant proteins.

As a rule, construction of plants–bioreactors for recombinant protein production entails large-scale screening of regions carrying the insertions of target genes regarding the highest yield of the recombinant protein and subsequent selection of the most optimal ones. When selecting the locus for insertion of a target gene, it is necessary to take into account its transcriptional activity as well as its association with vitally important genes whose malfunction can interfere with either important agricultural characteristics or viability of the organism in general. The CRISPR/Cas system is one of the most promising state-of-the-art tools allowing to enhance the biosynthesis of recombinant proteins. In particular, a prescreening of integration loci of a target expression cassette containing the genes coding for β-carotene has enabled some authors to create “golden rice,” which combines a high yield of the target product and valuable agricultural characteristics of the initial cultivar [118].

When assessing plant expression systems in terms of potential efficiency of the biosynthesis of recombinant proteins, it is necessary to take into consideration the utility of platforms based on cell suspension culture. A plant genome’s considerable part that is responsible for the function of the organism as a whole is not required in in vitro cell and tissue cultures [119,120]. Therefore, the risk of interference with the coordinated expression of own genes is lower after insertion of a transgene; accordingly, the selection of a proper insertion locus providing the most active protein synthesis remains the main task. On the one hand, housekeeping genes are most suitable for this purpose because their activity is maintained at a high level during the entire cell lifespan [121]. In particular, the delivery of a target gene called *dIFN*, coding for human γ-interferon, to the transcriptionally active region of the histone H3.3 gene with the help of CRISPR/Cas9 has helped to obtain monoclonal cell lines yielding a recombinant dIFN protein in the amount of >2% of TSP [122]. On the other hand, genome-editing technologies make it possible to reduce the screening of genome regions to a search for regions with a high yield of a recombinant protein using reporter genes. The genome regions characterized in terms of accumulation of a recombinant reporter protein (GFP) and the degree of disturbance of other genes (thereby causing a deterioration of, e.g., growth characteristics of cell culture) can serve as the targets for delivering a gene of interest with the help of the knock-in version of genome editing [121].

A low yield of the recombinant proteins produced in cultured plant cells is explainable not only by low expression in the plant system but also by the level of degradation of the target product. Various strategies are being devised to reduce proteolytic degradation, e.g., guiding of target proteins to cell compartments by attachment of signal peptides; coexpression of recombinant proteins and protease inhibitors [123]; creation of knockout mutations in genes coding for specific proteases [124]; elimination of protease-binding sites from the target genes; and RNA interference for suppression of protease synthesis [125]. These practices allow researchers to more than double a target protein’s production.

It is important to emphasize that additional studies on improving the efficiency of production stages are necessary to make protein expression commercially cost efficient via the optimization of either cell cultivation processes in bioreactors or the conditions of agrotechnical plant growth if plants will be used as a raw material for isolation of target recombinant products [126,127].

Along with the low yield, other serious challenges remain, including differences in glycosylation patterns between plant and mammalian cells. Specific features of a glycoform of a protein can influence its folding, aggregation, resistance to proteolytic degradation, solubility, and transport and can change its functional activity and immunogenicity [128]. Pharmaceutical value of recombinant proteins synthesized in plant cells and glycosylated according to the plant pattern is rather low; consequently, the ability of recombinant expression systems to efficiently and correctly glycosylate proteins in accordance with the human pattern is necessary to manufacture state-of-the-art pharmaceutical proteins. To date, new original strategies to change enzymatic pathways for the proteins synthesized in plant expression systems have been developed [74,75], and plants and plant cell lines having an optimized glycosylation profile matching the N-glycosylation pattern (characteristic of human proteins) are being constructed. This approach allows plant systems to produce authentic human glycoproteins (for instance, enzymes and antibodies) for the treatment of various diseases.

## 3. Specific Features of the Organization of the Plastid Transcriptional and Translational Machinery in the Plant Cell and Its Use as a Tool for the Biosynthesis of Recombinant Proteins

### 3.1. Structure of the Plastid Genome

Plastids, the best known of which are chloroplasts, are independently dividing semiautonomous organelles of the plant cell. Aside from chloroplasts, other kinds of plastids are known, such as proplastids, etioplasts, amyloplasts, and chromoplasts [129,130]. Plastids take part in photosynthesis but also perform other crucial functions, for example, synthesis of fatty acids, amino acids, vitamins, and pigments (this list is much longer) [131,132].

Plastids have a genome of their own (plastome), available for various genetic manipulations. On average, the plastome has a relatively small size (~150 kbp), although it may vary from 75 to 250 kbp depending on plant species [133]. Plastomes of angiosperms typically comprise approximately 130 genes with up to 80 coding for proteins, 40 genes of transport RNA (tRNA), and 4 genes of ribosomal RNA (rRNA). Plastids have their own transcriptional and translational machinery; however, most of it is encoded in the nuclear genome, synthesized in the cytoplasm, and subsequently transported to plastids [134]. Each plastid carries approximately 100 copies of the plastome. The ability of the plastome to autonomously replicate in the cell makes plastids a unique and very efficient biofactory. The structure of the plastid genome (an operon-like gene arrangement, bacterial-like RNA polymerase, and 70S ribosomes) reflects the origin of plastids from ancient free-living cyanobacteria. Plastids are also capable of post-translational mRNA processing and splicing [135]. Gene expression in plastids is regulated via mRNA stabilization and various transcription factors [136,137].

The elementary genome of plastids, i.e., the plant plastome, is thought to be a circular DNA molecule with densely arranged genes [138]. Nonetheless, the plastome can also exist in a simple linear form or a branched form, and the proportion of each form varies among different plant cell types [139]. In most higher plants, the structure of plastid DNA is rather conserved and contains two inverted repeats (IRa and IRb), which divide it into two unequal unique parts: the LSC (large single copy) region and SSC (small single copy) region [133,140]. Nevertheless, some legumes (pea and alfalfa) lack these repeats [133]. From an applied-research standpoint, this four-part structure is of great importance when transplastomic plants are constructed. Genetic constructs intended for the insertion into inverted repeats with the help of flanking sequences help to raise the rate of insertions via recombination.

At the suborganelle level, the plastid genome is arranged into compact nucleoprotein complexes (nucleoids), which are the structures associated with thylakoid membranes, and shows certain dynamics both of their number per plastid and of the plastome copy number per nucleoid, observable during leaf development. In particular, individual plastids in meristematic cells contain 2 to 6 nucleoids with 8 to 35 plastome copies in each, whereas a mature chloroplast contains 20–30 nucleoids with approximately 80–130 plastome copies in each. Quantitative estimates confirm continuous growth of the amount of plastid DNA per organelle and per cell during the development from a mesophyll post-meristematic/juvenile tissue to mature tissue; this process co-occurs with the differentiation of proplastids into chloroplasts [141]. As a rule, nucleoids are located on thylakoid membranes across the entire chloroplast, and this arrangement most likely plays an important role in their almost equal segregation during plastid division [142].

The plastid genome is characterized by both eukaryotic and prokaryotic traits. Approximately half of plastid genes are arranged in operons (as in prokaryotes); however, genes coding for functionally distinct proteins are located in plastid operons. Operons frequently have several promoters recognizable by different RNA polymerases. Unlike bacterial genes, 15 to 17 plastid genes carry introns, implying the presence of splicing (as in eukaryotes). In addition, an interesting characteristic of plastids is the editing of their transcripts. The last 20 years brought a considerably deeper insight into the mechanisms underlying expression of the plastid genome: first and foremost, transcription, which has turned out to be rather intricate [143,144].

### 3.2. Advantages of the Plastid Transcriptional and Translational Machinery for Recombinant Protein Biosynthesis

The semiautonomy of plastids and the presence of their own transcriptional and translational machinery (albeit to a considerable degree imported from the cytoplasm but weakly dependent on the nucleus) make them most attractive for biotechnology. Plastid transformation with a gene construct has a number of advantages over nuclear transformation. First, a recombinant gene commonly gets inserted into a random region of the nuclear genome via nonhomologous end joining; by contrast, this insertion mainly follows the homology-directed repair (HDR) mechanism in plastids, as in prokaryotic structures. The latter phenomenon provides precise insertion into a specified plastome region and enables the position effect, and overlapping with own plastome genes can be avoided. Second, the fact that genes in plastids are transcribed within operons guarantees that several genes of exogenous metabolic pathways can be inserted at once, thereby creating an artificial operon in plastids. Third, transplastomic plants contain a copy of each recombinant gene in each of approximately 100 elementary plastomes in all of ~100 chloroplasts within a cell, that is, up to 10,000 copies per cell rather than one or two copies per cell, as in the case of a nuclear transformation of a gene construct [145]. Because of two other specific features of plastids—a natural ability to express large amounts of gene products and almost complete absence of gene splicing and epigenetic modifications [146]—the amount of an accumulated recombinant protein expressed in plastids can be 10–100-fold larger as compared with the nuclear transformation [147,148], reaching up to 70% of plant TSP without any damage to plant viability [149]. Another important specific feature of plastids is their maternal inheritance. It guarantees stable expression of a gene even in the case of cross-hybridization of transplastomic plants and precludes any transfer of recombinant genes to other plants with pollen. Finally, each plastid is an isolated compartment. Although it is clear that many metabolites can partially leave a plastid for the cytosol, most of their amount and genetic material are protected by a double membrane and isolated from catabolic enzymes of the cytoplasm. Several vaccines synthesized in the plastids of *Chlamydomonas reinhardtii* and stored as freeze-dried cells at room temperature have been shown to retain their stability and activity after at least 0.5 and 1.5 years [150,151].

The first transformations of plastids, performed in *C. reinhardtii* and tobacco, date back to >25 years ago [152,153]. What followed was a slow and difficult increase in the range of plant species possessing transformed plastids. Nonetheless, transplastomic plants of tomato (*Solanum lycopersicon*), potato (*Solanum tuberosum*), lettuce (*Lactuca sativa*), soybean (*Glycine max*), cabbage (*Brassica oleracea* var. *capitata*), cauliflower (*Brassica oleracea* var. *botrytis*), sugar beet (*Beta vulgaris*), eggplant (*Solanum melongena*), carrot (*Daucus carota*), cotton (*Gossypium hirsutum*), and silver poplar (*Populus alba*) have been produced [154,155]. Quite recently, major success was achieved in the production of fertile transplastomic *Arabidopsis thaliana* plants [156]. A wide range of proteins for different purposes are now effectively produced in transplastomic plants, and several dozen proteins are currently synthesized in chloroplasts, including recombinant vaccines, monoclonal antibodies, and various enzymes for therapeutic and industrial applications [157,158,159,160].

Thus, transplastomic plants are most promising from the perspective of industrial production of a wide range of biologics. Nonetheless, several current problems limit the use of transplastomic plants as bioreactors. All these issues come down to two major ones, namely, a low probability of transformation of the plastid genome with a gene construct and the difficulties with the selection of homoplastomic and homoplastidic plants. The former is related to the need to construct special species-specific recombination cassettes. In addition, the methods used to deliver these cassettes into the plant cell play a considerable part in the enhancement of transformation efficiency. Finally, a key point regarding the increase in the magnitude of target protein biosynthesis is multistage selection by means of several antibiotics and the design of special breeding schemes to make the plant cells homoplastomic and homoplastidic.

### 3.3. Ways to Raise the Rate of Plastid Genome Transformation

The increase in the rate of integration of target genes into plastids remains a major challenge when transplastomic plant systems are used in biotechnology. This is a complex issue and is solvable at different levels, including the delivery of exogenous DNA into plastids, design of species-specific expression cassettes (optimally selected promoters, 5′ untranslated regions (UTRs), and 3′UTRs), selection of a site for integration into the plastome according to the HDR mechanism, and the use of a targeted genome-editing toolkit.

#### 3.3.1. Methods for Delivering Exogenous DNA to the Plastid Genome

Biolistics, i.e., the delivery of foreign DNA into plastids with the help of gold or tungsten microparticles, is employed the most frequently. This method is appropriate for cells of almost all plant species and lets an investigator vary many parameters of a gene gun, microparticles, and DNA concentration. Accordingly, it is possible to adjust the method to a particular structure of the plant cell membrane [146]. A recently designed novel strategy was proposed for plastid transformation with the help of chitosan-complexed single-walled carbon nanotubes (CS-SWNTs) using simple leaf infiltration [161]. The nanotubes are positively charged and thus can bind negatively charged plasmid DNA. During the infiltration into leaves, a DNA–SWNT conjugate can easily pass through the cell wall into mesophyll cells and then across the chloroplast double-membrane envelope [161]. In the chloroplast, DNA can be selectively released from the DNA–SWNT complex because of the pH difference between the cytosol and chloroplast medium. Weakly acidic pH of the cytosol in mesophyll cells keeps DNA in the complex with chitosan, while DNA is readily released into a weakly alkaline chloroplast medium. This approach gives considerable DNA accumulation in chloroplasts. Indeed, up to 35% of SWNTs reach chloroplasts and up to 88% of chloroplasts carry SWNTs [161,162]. This rate of delivery is by far higher than that of biolistic transformation of chloroplasts. This method has shown its efficiency in four plant species.

Another recently designed technique for the delivery of genetic material into plastids also takes advantage of simple leaf infiltration. This method is based on the formation of a complex between plant DNA (pDNA) and two signal peptides of 30–35 amino acid residues [163]. One peptide mediates transport across the cell wall and cytoplasmic membrane, and the other the transport along the intracellular membrane and cytoskeletal networks into chloroplasts. These two peptides electrostatically interact with pDNA and form a globule with a size of 170–200 nm. The plasmid–peptide complexes bind to chloroplast membranes 2 h after the infiltration to pass through them; thereafter, pDNA is released, and 24 h later, the corresponding products resulting from transient expression of reporter genes (luciferase or GFP) are detectable in chloroplasts. This method was successfully applied to *A. thaliana* and *N. benthamiana* chloroplasts, as well as to tomato and potato chromoplasts, leucoplasts, and amyloplasts. A comparison of the efficiency of this approach with biolistics has revealed that the latter gives only a few transformed chloroplasts per cell, whereas the former (the complex with peptides) succeeds in transforming 10–12 chloroplasts per cell. The luciferase activity in chloroplasts is three- to tenfold higher as compared with biolistics [163].

#### 3.3.2. Designing Vectors for Plastid Transformation

In contrast to the nuclear genome, where homologous recombination is hampered, the insertion of expression cassettes into the plastid genome primarily proceeds via the HDR mechanism, thereby ensuring precise targeting of a cassette to a specified plastome region [145,164]. The rate of homologous recombination is maximal when the expression cassette is flanked on both sides with ≥121-bp sequences homologous to the sequences at the integration site [165]. A correctly selected insertion site and elements of the expression cassette (promoter, 3′UTR, and 5′UTR) also substantially help to increase the rate of transgene integration and expression level.

##### Effects of Species Specificity of Spacer Sequences and of Selection of an Integration Site in the Plastome on the Insertion Rate and Expression of Recombinant Genes

Although the plastid genome in general is rather conserved, this mainly applies to coding regions. Intergenic spacers, however, into which recombinant genes are integrated, considerably differ among species. In particular, only 4 intergenic spacers out of 150 in the plastome are highly conserved among dicots [134], whereas monocots have none [166]. Nonetheless, even a single-nucleotide substitution in an exogenous flanking sequence of an expression cassette can dramatically lower the insertion rate in the case of the HDR mechanism. Within a species, intergenic spacers are as a rule identical [134]. Consequently, each species requires a species-specific vector with the flanking sequences sharing 100% homology with the intergenic spacer.

A position effect in its traditional sense is undetectable in the plastid genome; however, there are intergenic spacers with high and low transcriptional activities. That is why for a high expression level of a recombinant protein, it is crucial to select an efficient integration site. The highest expression of recombinant proteins in the overwhelming majority of cases has been achieved after integration into the transcriptionally active *trnI*/*trnA* spacer. Most transplastomic plants have been created via integration into this spacer, suggesting that this site holds promise for achieving high expression of recombinant proteins [167].

##### Using the CRISPR/Cas9 Genome-Editing System

The presence of a double-strand DNA break (DSB) in the region of an insertion site is key for effective integration of an expression cassette into the plastome according to the HDR mechanism [168]; this DSB can be induced by the CRISPR/Cas9 genome-editing system. Single-guide RNA (sgRNA), carrying a sequence homologous to the target region and guiding Cas9 endonuclease there, is used to guarantee precise positioning. Yoo et al. [169] have applied the CRISPR/Cas9 system for targeted integration of the *GFP* reporter gene into the *C. reinhardtii* plastome. The authors constructed a single plasmid harboring three expression cassettes: the first one carrying the *Cas9* gene, the second one encoding sgRNA, and the third one containing a donor cassette with the reporter *GFP* gene and genes coding for selective markers. The donor cassette was flanked with the sequences homologous to the regions of an integration site necessary for insertion according to the HDR mechanism as well as Cas9/sgRNA recognition sites, which ensured its excision from the plasmid. The plasmid was delivered to chloroplasts by biolistics. In this experiment, 2 of the 20 produced transformants carried the reporter gene in the specified region of the plastome. Of note, there were no cases of targeted transformation when the plasmid without the *Cas9* gene was used; this finding means that a DSB is necessary to guarantee the insertion in accordance with the HDR mechanism [169]. An analogous gene construct with the genome-editing system has also been employed to transform tobacco chloroplasts. The authors demonstrated that the targeted DSBs produced by the Cas9/sgRNA complex raise the rate of chloroplast transformation by 6–10-fold [170].

##### Effects of Promoters and 5′UTR and 3′UTR Regulatory Elements on the Expression of Recombinant Genes in Plastids

Plastids of higher plants contain RNA polymerases of two types—the plastid-encoded RNA polymerase (PEP) encoded by the plastome and nucleus-encoded RNA polymerase (NEP) encoded in the nuclear genome—which strongly distinguish the plastid transcriptional machinery from the nuclear one. PEP and NEP differ in the structure of the promoters they recognize [171]. Generally, PEP-specific promoters are considerably stronger as compared with NEP-specific ones, and the majority of most intensively transcribed plastid genes are under the control of PEP-specific promoters, which is also a distinct feature of the plastid transcriptional machinery [172]. Accordingly, the use of PEP promoters is preferable for the biosynthesis of recombinant proteins. The strongest promoter in higher-plant plastids, *Prrn*, controls the ribosomal RNA operon, and in particular, *Prrn* was used when many efficient expression cassettes were designed. Because rRNA is not translated, high expression of a recombinant protein requires that *Prrn* be supplemented with the corresponding signal for translation initiation [173]. The *psbA* operon promoter is also promising for plastid transformation; this promoter is transcribed intensively, and moreover, its transcripts are actively translated by the plastid translational machinery, which in many cases gives higher expression levels as compared with the *Prrn* promoter [174]. Along with the plastid promoters, strong bacterial or mitochondrial promoters are also utilized for controlling expression of recombinant genes. Although they are recognized by the plastid transcriptional machinery, they are actually weaker than the PEP-specific promoters [175].

Gene expression in plastids is mainly regulated at the translational level [176]; therefore, it is not surprising that correct selection of 5′UTRs and 3′UTRs is of paramount importance to attain a high level of target gene expression in plastids. Not only plastid 5′UTRs but also several exogenous 5′UTRs (mostly bacterial but also synthetic) have been tested for the expression level of reporter genes [177]. The 5′UTR of the T7 bacteriophage gene 10 has emerged as one of the strongest factors [178]. In combination with plastid *Prrn*, it yields one of the highest levels of recombinant protein accumulation [178,179]. The promoter/5′UTR of the plastid *psbA* gene, coding for the most intensively translated plastid protein, gives very high transgene expression [180]. The expression activity drastically drops when exogenous *psbA* is used, e.g., lettuce *psbA* in tobacco plastids or vice versa. The differences between the tobacco and lettuce 5′UTR sequences considerably decrease 5′UTR affinity for RNA-binding proteins [174]. This is another observation suggesting that high expression levels of recombinant proteins in plastids require construction of species-specific vectors.

The 3′UTRs of plastid mRNAs form stable loops in their secondary structure, which protect mRNA from rapid degradation and allow for its accumulation [181]. Numerous duties of plastid 3′UTRs suggest that the choice of 3′UTRs influences the level of mRNA accumulation, but their contribution to recombinant protein expression is rather limited [182]. This finding is consistent with the observation that gene expression regulation in plastids is mostly confined to the translational level, and the mechanisms underlying the regulation of plastid translational machinery can withstand even high levels of mRNA accumulation [182,183].

Thus, the expression level of a target gene in transplastomic plants strongly depends on many factors: on correct selection of the insertion site, a promoter, and regulatory 5′UTRs and 3′UTRs to name the most important ones. Table 4 illustrates the variation of accumulation levels of recombinant proteins; note that the first six rows in Table 4 represent an optimal combination of regulatory elements and an integration site (*trnI*/*trnA*).

### 3.4. Methods for Constructing Homoplastomic and Homoplastidic Plants

**Figure 2:**
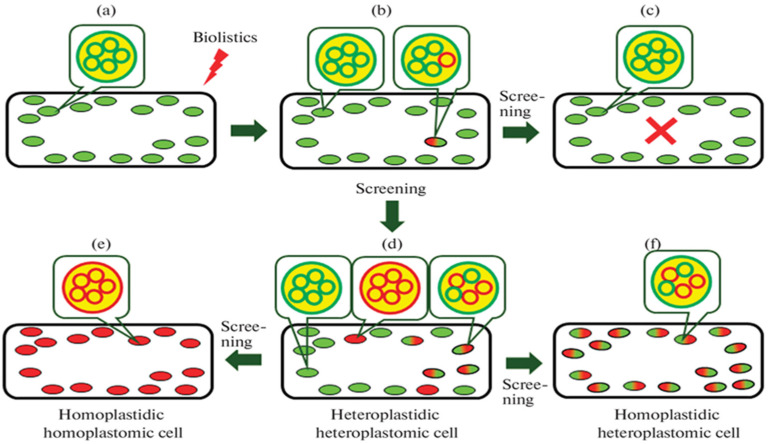
Reorganization of the plastid genome after selection on selective media to achieve homoplastomy and homoplastidic status. (**a**) Introduction of a genetic construct into plastids. (**b**) Insertion of one or more copies of a transgene into the plastid genome via homologous recombination. (**c**) Cells without insertions extinct. (**d**–**f**) Outcomes of cell selection on a medium with a selective agent (usually, antibiotic spectinomycin alone or with streptomycin). Green ovals within the cell boundaries are native chloroplasts with the untransformed genome; two-color ovals: chloroplasts containing transgenic and nontransgenic copies of the genome (heteroplastomic chloroplasts); red ovals: chloroplasts containing only transformed copies of the genome (homoplastomic chloroplasts); callouts show individual chloroplast nucleoids containing only nontransgenic copies of the genome (green circles), nucleoids containing a mixture of transgenic and nontransgenic copies of the genome (a combination of green and red circles), and nucleoids containing only transgenic copies of the genome (red circles).

One of the major problems with constructing transplastomic plants offering a high recombinant protein expression level is that it is difficult to obtain the cells in which the plastomes of all plastids carry the transferred gene and which can be subsequently used to regenerate homoplastomic and homoplastidic plants. During the last 20 years, techniques for producing homoplastomic and homoplastidic genes have not improved much and still rely on multiage selection involving two or more selection factors. Figure 2 shows a general scheme of plastid genome transformation via multistage selection on selective media. The ideal situation resulting in the highest level of target gene expression is the construction of homoplastomic and homoplastidic cells (Figure 2e). A recent new trend in this selection scheme is the transformation of proplastids of meristematic and etiolated callus tissues initially carrying a few plastids with a small number of plastomes rather than mature chloroplasts.

#### 3.4.1. Using Meristematic and Etiolated Callus Tissues to Transform Plastids

One mature chloroplast can carry >100 plastomes. In the process of transformation, recombinant genes are inserted into only one or a few plastomes, while most of plastomes remain intact. Consequently, it is reasonable to transform the cells and tissues that contain a small number of plastids with a few plastomes. Meristematic apical tissues house a small number of proplastids and immature chloroplasts each carrying approximately 10 plastomes, while mature cells of the leaf mesophyll contain up to 2600–3300 copies per cell [185,186]. In etiolated cell and callus cultures, chloroplasts dedifferentiate into proplastids; the number of plastomes they harbor drastically diminishes, as does the number of plastids per cell [186,187,188]. The use of meristematic tissues and etiolated cell cultures for transformation considerably decreases the number of plastomes that remain intact, thereby substantially facilitating the subsequent selection for homoplastomy and a homoplastidic state. In particular, callus cell transformation permits construction of transplastomic *A. thaliana* and sugar cane (*Saccharum officinarum*) plants, although the homoplastomy and homoplastidic status of such plants in some cases remains questionable [189,190,191].

#### 3.4.2. Selection and Selective Markers for Achieving Homoplastomy and a Homoplastidic State

The majority of cell plastomes remain intact after the transformation; hence, to attain homoplastomy and a homoplastidic state, it is necessary to perform multistage selection for one or two specific markers. Different genes conferring resistance to different antibiotics are used to obtain transplastomic plants. Most popular antibiotics, such as spectinomycin, streptomycin, kanamycin, and chloramphenicol, are selective inhibitors of protein synthesis on prokaryotic-type ribosomes (70S) and have no effect on protein synthesis involving 80S ribosomes in the cytoplasm. These antibiotics are usually applied at initial selection stages, when only a few plastomes carry selective markers [192]. The expression of genes of neomycin phosphotransferase (*nptII*) or aminoglycoside-3′-phosphotransferase (*aphA6*) provides kanamycin resistance [193,194]. The selective marker most widely used in plastid transformation is the gene encoding streptomycin 3′-adenylyltransferase (*aadA*), rendering plastids resistant to two antibiotics: streptomycin and spectinomycin [195,196]. Spectinomycin binds to plastid ribosomes and blocks protein synthesis [197]. The selection of media containing these two antibiotics (streptomycin + spectinomycin) allows for separation of the transplastomic clones from frequently emerging spontaneous mutants resistant to spectinomycin [192]. Chloramphenicol resistance, conferred by the gene of bacterial chloramphenicol acetyltransferase (*CAT*), is less pronounced as compared with the kanamycin or spectinomycin resistance; however, its advantage is the absence of spontaneous stable mutants [198]. The selection of transplastomic clones by resistance to 4-methylindole 7-methyl-DL-tryptophan (anthranilate synthase gene, *ASA2*) [199], or betaine aldehyde (betaine aldehyde dehydrogenase gene, *badh*) [200], has also been reported to be effective.

At the early stages of selection, when the transformed plastomes are still few, it is necessary to keep in mind the phenomenon of gene conversion, which is widespread in plastids. If a target gene is not tightly linked to the selective marker used, it may be lost despite preservation of resistance to the selective marker, because the majority of template plastomes lack the target gene [201]. Accordingly, for preventing the loss of the target gene, it is strongly recommended to subject the transplastomic plants to additional selection cycles in order to ensure their homoplastomic state and to analyze a large number (tens) of regenerants in each regeneration cycle by either a dot blot or PCR [146].

At the final stages of the selection, when the copy number of the transformed plastome goes up, it is expedient to employ secondary selective markers, such as *bar* (conferring resistance to herbicides phosphinothricin and glyphosate [202]), as well as genes providing resistance to sulfonylurea, pyrimidinylcarboxylate [203], and diketonitrile [204].

A recently proposed new original technique helps to efficiently achieve homoplastomy when plastids are transformed [205]. This methodology is based on the *barnase–barstar* gene pair from *Bacillus amyloliquefaciens* [206]. The *barnase* gene codes for a ribonuclease with a cytotoxic effect, while the product of the *barstar* gene is its specific inhibitor [207]. The authors fused the *barnase* gene with a signal guiding it to plastids and placed it under the control of a β-estradiol-inducible system [208]. The final construct was inserted into the tobacco nuclear genome, and the plastids were transformed with the vector carrying the *barstar* gene and *GFP* reporter gene. Estradiol induction permitted only the *barstar*-transformed plastids to survive; thus, the investigators rapidly obtained homoplastomic and homoplastidic tobacco plants [205].

### 3.5. Using Transplastomic Plants for Recombinant Protein Synthesis

A wide range of proteins for different applications is now successfully synthesized in transplastomic plants. In particular, over 40 therapeutic proteins are synthesized in *C. reinhardtii* chloroplasts, including antigens of various pathogens, subunit vaccines, enzymes, interleukins, monoclonal antibodies, growth hormones, protein adjuvants, and immunotoxins. For the most part, this list includes single-subunit proteins requiring the introduction of only one gene; however, there are cases of assembly of light and heavy chains of human monoclonal antibodies directly in chloroplasts [184,208]. The enzymes actively used in the production of detergents and in the textile industry, e.g., cellulases, lipases, and mannases, are synthesized in tobacco and lettuce chloroplasts. These enzymes produced in chloroplasts are stable in extracts of fresh leaves without added protease inhibitors, and in contrast to their liquid commercial analogs, do not need to be stored or transported in a refrigerator and can retain their activity in the long term at room temperature in the form of a powder prepared from freeze-dried leaves [158].

A wide variety of different cellulases, ligninases, and pectinases, necessary to produce biofuel, can be synthesized in tobacco leaves (over 20 enzymes) in the amount of up to 5–40% of plant TSP [159]. An artificial operon comprising three additional genes of the tocochromanol pathway (a limiting stage in vitamin E biosynthesis) has been introduced into tobacco and tomato plastomes, resulting in a 10-fold increase in the XYZ content of plants [160]. In that study, to examine the feasibility of enhancing vitamin E accumulation in plants, plastids were transformed with two kinds of synthetic operons. The first version was of the bacterial type with the expression of three genes coding for enzymes of the tocochromanol pathway controlled by a single promoter and individual coding sequences separated by two intergenic spacers from endogenous operons of the chloroplast genome. In this case, tocopherol expression caused only a moderate (~1.7-fold) increase in tocopherol accumulation. Likely reasons were low stability and inefficient translation of the polycistronic mRNA. In the other synthetic operon, individual genes were separated by a recently identified mRNA element known as an intercistronic expression element [209], triggering processing of polycistronic transcripts into stable translated monocistronic mRNAs. This approach resulted in 10-fold greater accumulation of tocochromanol. Thus, the insertion of an intercistronic expression element between genes triggers the processing of polycistronic transcripts into stable translatable monocistronic units, thereby driving their effective expression. Consequently, the synthetic operons designed in this way can help to take advantage of new biochemical pathways in plants [160]. A recent review [184] comprehensively describes the lists of species of transplastomic plants and the proteins of interest synthesized in these plants.

## 4. The Mitochondrial Genome

Mitochondria are powerhouses of the cell and implement oxidative phosphorylation processes. Similar to plastids, mitochondria are endosymbionts of a pro-eukaryotic cell, have their own genome, and their own transcriptional and translational machinery. Unlike the plastid genome (represented by approximately 100 plastome copies), the genome of mitochondria (mitogenome) is not so over-replicated in most plant species. Animal mitochondrial genomes are rather small (~15 kbp); on the contrary, the plant mitogenome is considerably larger and varies in size by two orders of magnitude depending on the species: from 66 kbp in the hemiparasitic mistletoe (*Viscum scurruloideum, Santalaceae*) [210] to 11,300 kbp in the sand catchfly (*Silene conica, Caryophyllaceae*) [211]. On average, mitogenome size in terrestrial plants (300–400 kbp) is two- to threefold longer as compared with the plastid genome (100–200 kbp) [212]. In contrast to the plastid genome, coding genes in the mitogenome are much less densely arranged, and their total number is only 3–66 [213].

### 4.1. Specific Features of the Organization and Function of the Plant Mitochondrial Genome

As compared with the conserved plastid genome and compact animal mitochondrial genome, the plant mitochondrial genome possesses several unique specific features. Cases of extensive homologous recombination of plant mitochondrial DNA (mtDNA) are frequent, underlying its propensity for reorganization. The structure and size of plant mtDNA considerably changes because of intensive proliferation of mobile genetic elements, an increase in introns, and insertions of foreign DNA (nuclear, plastid, viral, or bacterial) [211]. Along with a single circular chromosome, as in *A. thaliana* [214], many plant species contain several circular or even linear chromosomes in their mitochondrial genome. For instance, cucumber (*Cucumis sativus*) has three mitochondrial chromosomes with lengths of 1156, 84, and 45 kbp [215]. Another example is *S. conica*, the 11,300 kbp genome which is composed of 128 circular molecules replicating independently of one another [211]. A key feature of the organization of plant mitochondrial genomes is that the noncoding rather than coding sequences constitute its main body. The copy number of the plant mitogenome is considerably lower as compared with animals and pronouncedly varies depending on the developmental stage or analyzed tissue. In particular, the maximum observed copy number of the *A. thaliana ATP1* gene is approximately 280 copies per cell, being lower than the average number of mitochondria per cell (approximately 500). This observation suggests that plant mitochondria can carry a partial mitochondrial genome or even be devoid of it [216]. The plant mitochondrial genome contains direct and inverted repeats, which are involved at a high rate in the recombination and rearrangement of mtDNA (e.g., 6.5 and 4.2 kbp repeats in *Arabidopsis* mtDNA) [217]. Recombination also involves short repeats but at a considerably lower rate. In general, plant mtDNA in vivo is most likely represented by a set of recombination intermediates, concatemers, subgenomic circular molecules, and plasmids. A certain part of plant mtDNA exists in a single-stranded form (as observed in *Chenopodium album*) [218].

The plant mitochondrial genome has the form of a nucleoid, i.e., nucleoprotein particles anchored to the inner mitochondrial membrane. The role of the nucleoid is mtDNA compaction and regulation of its metabolism and transcriptional activity, while its external components are involved in signaling systems. Note that nucleoids of plant mitochondria contain many proteins binding single-stranded rather than double-stranded DNA. Some proteins of the nucleoid are double-targeted and function in plastids as well. All nucleoid proteins of *A. thaliana* mitochondria are encoded in the cell nucleus [219]. Unlike plastids, which carry two types of RNA polymerases (PEPs (encoded in the plastid genome) and NEPs (encoded in the nucleus)), mitochondria of almost all eukaryotes have lost bacterial-type polymerases (PEPs), and hence NEPs exclusively participate in transcription (some of them function in plastids, too) [220].

### 4.2. Prospects of Transcriptional and Translational Machinery of Plant Mitochondria for Recombinant Protein Biosynthesis

Long-term efforts to implement genetic transformation of mitochondria have not been fruitful: so far, only mitochondria of the yeasts *Saccharomyces cerevisiae*, *C. reinhardtii* [221], and *Candida glabrata* [222] have been successfully transformed by biolistics. The success of genetic transformation of the mitochondria of these lower eukaryotes is attributable to the specific features of their cell metabolism, which are absent in higher eukaryotes. As for higher plants, the main, albeit unsolved, problem hindering the creation of such a system is the absence of a selection strategy for cells with genetically transformed mitochondria. High antibiotic sensitivity of mitochondrial respiratory chain complexes prevents the use of antibiotics for the selection of transmitochondrial cells or plants.

In the past few years, significant advances have been made in terms of the editing of plant mitogenomes. A system has been developed that can quite effectively modify mitogenomes of land plants with the help of transcription activator-like effector nucleases having mitochondria-targeting localization signals (mitoTALENs) to induce DSBs in targeted mitochondrial genes. By agrobacterial nuclear transformation of rice and rapeseed with this mitoTALEN system, knockouts of cytoplasmic male sterility mitochondrial genes have been obtained in these plants [223]. The floral deep transformation method with mitoTALENs made it possible to obtain a knockout of two genes of ATP synthase [224], and with mitochondria-targeting TALEN-based cytidine deaminase, this approach can replace C:G with T:A pairs in a given region of the mitogenome of *A. thaliana* [225]. Nevertheless, no events of integration of foreign recombinant genes into the mitogenome have been recorded so far. Be that as it may, this field of gene engineering is developing rapidly, and it is likely that in a few years new tools for editing mitogenomes in the knock-in format based on CRISPR/Cas technologies will be devised.

Thus, plant mitochondria remain the most appealing as the expression system for producing recombinant proteins (and even possibly more attractive than chloroplasts) if we take into account certain fundamental specific features of the genetic system of these organelles. Among the most important of these features is that mitochondria of higher organisms are able to take up (import) DNA, thereby creating favorable conditions for in vivo delivery of genetic constructs to mitochondria during biotechnological manipulations. The mitochondrial plasmids present in the mitogenome of some plant species and their simpler structural–functional organization make them the most suitable for the insertion of target genes. High tolerance of the mitochondrial genome to incorporation of foreign DNA (as evidenced by DNA fragments of nuclear, chloroplast, viral, and unknown origins in the mitogenome) also improves the chances of obtaining an efficient producer. Several years of research aimed at clarifying the functional role of the above-mentioned specific features of the genetic system of plant mitochondria (and at assessing their potential in the relevant biotechnological applications) strongly indicate their promising prospects [226,227,228]. As shown by some authors, it is feasible to insert a reporter gene (a *GFP* fragment) into the genome of isolated potato, maize, or tobacco mitochondria [229]. Expression of a target gene in mitochondria is undoubtedly preferable to its expression in the nuclear genome, because the production and accumulation of the recombinant protein is confined to the mitochondrial matrix. Consequently, the yield of the target protein is improvable via a decrease in its proteolysis and in its potential cytotoxicity. The use of a polyploid mitochondrial genome to express recombinant proteins offers advantages in terms of both the yield of the target product and lower probability of target gene silencing. As reported in one paper, the activity of mtDNA import considerably increases when a transported linear DNA molecule carries terminal inverted repeats, similarly to linear mitochondrial plasmids and transposons [227]. Recently, the possibility of DNA import into mitochondria was shown in *A. thaliana* protoplasts [230]. Thus, based on the above results, we can expect the development of a strategy in the near future that allows transfection of protoplasts with constructs carrying specific sequences that ensure their insertion into the mitogenome [229] and transcription of the target gene. With subsequent regeneration of the cell wall in these protoplasts, it becomes possible to select the transformed cells and either maintain them in cell culture or regenerate whole plants.

## 5. Conclusions

Thus, all three components of the plant genome—the nuclear genome, plastid genome, and mitogenome—are of appreciable interest for biotechnology, namely for the synthesis of recombinant proteins of all kinds: from protein vaccines to industrial enzymes. Each genome has its own molecular transcriptional and translational machinery providing different expression levels of recombinant proteins. Each of the three genomes has its own specific structural–functional features, requires different approaches to the design of transformation vectors, and offers distinct advantages and disadvantages.

So far, the nuclear genome remains the most popular for synthesizing recombinant proteins. First and foremost, this is explained by the relative simplicity of its transformation with a genetic construct; however, the main shortcoming of this genome is still low expression of recombinant proteins. On the other hand, the use of the nuclear–cytoplasmic transcriptional and translational machinery permits stable or transient expression of target genes, thereby helping to relatively rapidly produce recombinant proteins in small amounts. In addition, the same transcriptional and translational machinery can be successfully utilized in a cell-free medium, ensuring rapid synthesis of a target protein in appreciable quantities. The cells with the transformed nuclear genome can next be used to obtain both whole transformed plants and cell suspension cultures, making it possible to quickly and relatively inexpensively produce target proteins. During nuclear transformation, the target gene usually gets inserted into a random genome region that may prove to be inappropriate because of low transcriptional activity and a high probability of splicing. That is why it is reasonable to start with detection of the genome regions displaying a high transcriptional activity and a low risk of insertional damage to vitally important genes. For this purpose, one can employ reporter genes followed by targeted insertion of the genes of interest into the advantageous regions using the CRISPR/Cas9 genome-editing system.

There are no doubts that plastid transformation opens up a new field of plant biotechnology and bioengineering. Transplastomic plants have numerous advantages over nuclear transformants, including a potentially high yield of the target protein, feasibility of targeted insertion of the gene of interest into the prespecified region of the plastome via homologous recombination, and the possibility of transferring blocks of genes of whole metabolic pathways with the help of artificially constructed operons. The placing of target genes into plastids almost completely precludes their silencing and reduces the position effect to a minimum. Additionally, transplastomic plants are more reliable in biosafety terms because pollen does not carry plastids to other plants. Therefore, transplastomic plants may cause breakthroughs in biotechnology: from creation of new therapeutics to large-scale synthesis of industrial enzymes and biofuels. Nevertheless, there are still serious problems to be resolved, the main of which are the low rate of plastid transformation and the difficulties with selection of homoplastomic and homoplastidic plants. Tentative solutions are already clear. The first challenge requires the development of new systems for the delivery of foreign genes to plastids and fine design of transformation vectors. The difficulties with achieving homoplastomy can be overcome via basically new selection systems (for example, *barnase*/*barstar*) and the use of meristematic and dedifferentiated cells with a few plastomes for transformation.

The transformation of the plant mitochondrial genome with a genetic construct looks in a way even more attractive as compared with the plastid genome, largely because of (i) the natural competence of mitochondria for DNA import (which enhances the transformation), (ii) the presence of plasmids in mitogenome, (iii) high genome abundance, and (iv) a large number of mitochondria per cell. Additionally, the double membrane of mitochondria protects the proteins from cytosolic proteases. Unfortunately, the transformation of mitochondria has not been implemented yet, primarily owing to the absence of an efficient strategy for the selection of transformed mitochondria.

## Figures and Tables

**Table 1:** Commercial pharmaceutical proteins with stable expression in plants.

Plant Species	Recombinant Protein	Productivity	Reference
*Nicotiana tabacum*	Recombinant human erythropoietin (rhEPO)	66.75 pg/mg TSP	[21]
*Physcomitrella patens*	α-Galactosidase A	0.5 mg/mL TSP	[22]
*N. benthamiana*	Human glucocerebrosidase	68 μg/g FW	[23]
*Salvia miltiorrhiza*	Human acidic fibroblast growth factor 1 (FGF-1)	272 ng/g FW	[24]
*Helianthus annuus*	Lumbrokinase (LK)	5.1 g/kg seed	[25]
*N. tabacum*	Hydrophobin	16.5% of TSP	[26]
*Oryza sativa*	Human growth hormone (hGH)	57 mg/L of medium TSP	[27]
*Glycine max*	Fibroblast growth factor (bFGF)	2.3% of TSP	[28]
*N. tabacum*	Placental alkaline phosphatase (SEAP)	3% of TSP	[29]
*O. sativa*	Human serum albumin, epidermal growth factor, recombinant lactoferrin, basic fibroblast growth factor, insulin-like growth factor-1 LR3, lysozyme, vascular endothelial growth factor, α-1 antitrypsin, keratinocyte growth factor, and fibronectin	–	[30]
*O. sativa*	Transferrin and lysozyme	–	[31]
*N. tabacum*	B lymphocyte activating factor and bone morphogenic protein 7		
*Zea mays*	TrypZean^®^		
*Hordeum vulgare*	Leukemia inhibitory factor	–	[32]
	Human growth factors, cytokines, MESOkine (animal-like growth factors)	–	[33]
*N. tabacum*	Monoclonal antibody 2G12	10–25 μg/g FW	[34]
	Anti-HBsAg	6.5 mg/g seeds	[35]
*Z. mays*	Monoclonal antibody 2F5	0.61 ± 0.28 μg/g seed extract	[36]

Abbreviations. TSP: total soluble protein, FW: fresh weight.

**Table 2:** Commercial nonpharmaceutical proteins produced in plants.

Product	Company	Application	Plant Species	Reference
Trypsin, avidin, and endo-1,4-β-d-glucanase	ProdiGene/Sigma-Aldrich (St. Louis, MO, USA)	Technical reagents	Maize (seeds)	[43]
Cellobiohydrolase I	Infinite Enzymes/Sigma-Aldrich (St. Louis, MO, USA)	Technical reagent	Maize (seeds)	
Growth factors, cytokines, thioredoxin, and TIMP-2	Agrenvec (Madrid, Spain)	Research reagents	Tobacco (leaves, transient expression)	[44]
Growth factors and cytokines	ORF Genetics (Kópavogur, Iceland)	Research reagents	Barley (seeds)	[45]
Epithelial growth factor	Sif Cosmetics (Kópavogur, Iceland)	Cosmetics	Barley (seeds)	[46]
Albumin, lactoferrin, lysozyme, transferrin, and insulin	Ventria Bioscience/InVitria (Fort Collins, CO, USA)	Research reagents	Rice (seeds)	[47]
Aprotinin	Kentucky BioProcessing (Owensboro, KY, USA)	Research reagent	Tobacco (leaves, transient expression)	[48]
Collagen	CollPlant (Rehovot, Israel)	Research reagent, tissue culture, and therapeutic	Tobacco	[49]
Trypsin, enterokinase, growth factors, and cytokines	Natural Biomaterials (Wanju-gun, Republic of Korea)	Research reagents and cosmetic ingredients	Rice (seeds)	[50]
Antibody	Center for Genetic Engineering and Biotechnology (Havana, Cuba)	Reagent for purification of hepatitis B vaccine	Transgenic tobacco	[51]
α-Amylase	Syngenta (Wilmington, DE, USA)	Reagent for bioethanol production	Maize seeds	[52]
Phytase	Origin Agritech (Beijing, China)	Feed	Maize seeds	[53]
Growth factors	NexGen (Suwon, Republic of Korea)	Tissue culture reagent	Tobacco leaves, transient expression	[54]

**Table 3:** Recombinant proteins synthesized in cultured plant cells.

Plant	Protein	Productivity	Reference
*O. sativa*	Human cytotoxic T-lymphocyte antigen 4-immunoglobulin (hCTLA4Ig)	43.7 mg/L	[63]
	Human granulocyte macrophage-colony stimulating factor (hGM-CSF)	31.7 mg/L	[64]
	Cyclic citrullinated peptide (CCP) antibody	22.9 mg/L	[65]
	Human pepsinogen C (hPGC)	18 mg/L	[66]
	Glucocerebrosidase (GDC)	–	[67]
*D. carota*	β-Glucocerebrosidase and α-galactosidase A	–	[68]
*N. tabacum* (BY-2)	Actin inhibition resistance (AIR) DNase and antitumor necrosis factor	–	[68]
	IgG4	25 mg/L	[61]

**Table 4:** Variation of expression of several recombinant proteins depending on the insertion site, promoters, and regulatory regions (according to [184] with some updates).

Insertion Sites	Promoter/5′UTR/Terminator (3′UTR)	Expression Efficiency, % of TSP
*trnI*/*trnA*	P*psbA*/T*psbA*	32–38 and 17–26
*trnI*/*trnA*	P*rrn*/T*psbA*	45.3
*trnI*/*trnA*	T*rbcL*	10.0
*trnI*/*trnA*	P*rrn*/T*psbA*	0.85–1.0
*trnI*/*trnA*	P*psbA*/T*psbA*	5.16–9.27
*trnI*/*trnA*	P*psbA*/T*psbA*	0.2–6.0
*trnV*/*rps12*, *7*	*Prrn*/T*rbcL*	>7.0
*rbcL*/*accD*	P*psbA*/*rbcL 3*′ T*rbcL*	5.0
*rps7*, *12*/*trnV*	P*rrn*/T*7g10*/T*rps16*	>10.0
*rbcL*/*accD*	P*rrn*/T*psbA*	2.0–3.0
*trnfM*/*trnG*	P*rrn*/T*7g10*/*rbc3*′	0.8–1.6

## References

[1] Plant-based Biologics and Expression Systems Global Market Report 2022: Profiles of Medicago, Leaf Expression Systems, Eleva, iBIO, PlantForm, G+Flas Life Sciences, Kentucky Bioprocessing and Angany. https://finance.yahoo.com/news/plant-based-biologics-expression-systems-101500030.html.

[2] Burnett M.J.B., Burnett A.C. (2020). Therapeutic recombinant protein production in plants: Challenges and opportunities. Plants People Planet..

[3] https://www.researchandmarkets.com.

[4] Gill K.S., Gupta P.K., Varshney R.K. (2004). Gene Distribution in Cereal Genomes.

[5] Dong O., Ronald P. (2019). Genetic Engineering for Disease Resistance in Plants: Recent Progress and Future Perspectives. Plant Physiol..

[6] Gatehouse A.M.R., Ferry N., Edwards M.G., Bell H.A. (2011). Insect-resistant biotech crops and their impacts on beneficial arthropods. Philos. Trans. R. Soc. Lond. B Biol. Sci..

[7] Wani S.H., Dutta T., Neelapu N.R.R., Surekha C. (2017). Transgenic approaches to enhance salt and drought tolerance in plants. Plant Gene.

[8] Maghari B.M., Ardekani A.M. (2011). Genetically modified foods and social concerns. Avicenna J. Med. Biotechnol..

[9] Paul J.Y., Khanna H., Kleidon J., Hoang P., Geijskes J., Daniells J., Zaplin E., Rosenberg Y., James A., Mlalazi B. (2017). Golden bananas in the field: Elevated fruit pro-vitamin A from the expression of a single banana transgene. Plant Biotechnol. J..

[10] Zhang C., Wohlhueter R., Zhang H. (2016). Genetically modified foods: A critical review of their promise and problems. Food Sci. Hum. Wellness.

[11] Liao P., Chen X., Wang M., Bach T.J., Chye M.L. (2018). IMPROVED fruit α-tocopherol, carotenoid, squalene and phytosterol contents through manipulation of Brassica juncea 3-hydroxy-3-methylglutaryl-coa synthase1 in transgenic tomato. Plant Biotechnol. J..

[12] Kumar S., Palve A., Joshi C., Srivastava R., Rukhsar K. (2019). Crop biofortification for iron (Fe), zinc (Zn) and vitamin A with transgenic approaches. Heliyon.

[13] Buyel J.F., Twyman R.M., Fischer R. (2017). Very-large-scale production of antibodies in plants: The biologization of manufacturing. Biotechnol. Adv..

[14] Chan H.-T., Daniell H. (2015). Plant-made oral vaccines against human infectious diseases—Are we there yet?. Plant Biotechnol. J..

[15] Clarke J.L., Waheed M.T., Lossl A.G., Martinussen I., Daniell H. (2013). How can plant genetic engineering contribute to cost-effective fish vaccine development for promoting sustainable aquaculture?. Plant Mol. Biol..

[16] Kolotilin I., Topp E., Cox E., Devriendt B., Conrad U., Joensuu J., Stöger E., Warzecha H., McAllister T., Potter A. (2014). Plant-based solutions for veterinary immunotherapeutics and prophylactics. Vet. Res..

[17] MacDonald J., Doshi K., Dussault M., Hall J.C., Holbrook L., Jones G., Kaldis A., Klima C.L., Macdonald P., McAllister T. (2015). Bringing plant-based veterinary vaccines to market: Managing regulatory and commercial hurdles. Biotechnol. Adv..

[18] Schillberg S., Finnern R. (2021). Plant molecular farming for the production of valuable proteins—Critical evaluation of achievements and future challenges. J. Plant Physiol..

[19] Park K.Y., Wi S.J. (2016). Potential of Plants to Produce Recombinant Protein Products. J. Plant Biol..

[20] Zagorskaya A.A., Deineko E.V. (2021). Plant-Expression Systems: A New Stage in Production of Biopharmaceutical Preparations. Russ. J. Plant Physiol..

[21] Gurusamy P.D., Schafer H., Ramamoorthy S., Wink M. (2017). Biologically active recombinant human erythropoietin expressed in hairy root cultures and regenerated plantlets of *Nicotiana tabacum* L.. PLoS ONE.

[22] Lenders M., Brand E. (2021). Fabry disease: The current treatment landscape. Drugs.

[23] Limkul J., Misaki R., Kato K., Fujiyama K. (2015). The combination of plant translational enhancers and terminator increase the expression of human glucocerebrosidase in Nicotiana benthamiana plants. Plant Sci..

[24] Tan Y., Wang K.Y., Wang N., Li G., Liu D. (2014). Ectopic expression of human acidic fibroblast growth factor 1 in the medicinal plant, Salvia miltiorrhiza, accelerates the healing of burn wounds. BMC Biotechnol..

[25] Guan C., Du X., Wang G., Ji J., Jin C., Li X. (2014). Expression of biologically active anti-thrombosis protein lumbrokinase in edible sunflower seed kernel. J. Plant Biochem. Biotechnol..

[26] Reuter L.J., Bailey M.J., Joensuu J.J., Ritala A. (2014). Scale-up of hydrophobin-assisted recombinant protein production in tobacco BY-2 suspension cells. Plant Biotechnol. J..

[27] Kim T.G., Baek M.Y., Lee E.K., Kwon T.H., Yang M.S. (2008). Expression of human growth hormone in transgenic rice cell suspension culture. Plant Cell Rep..

[28] Ding S.H., Huang L.Y., Wang Y.D., Sun H.C., Xiang Z.H. (2006). High-level expression of basic fibroblast growth factor in transgenic soybean seeds and characterization of its biological activity. Biotechnol. Lett..

[29] Komarnytsky S., Borisjuk N.V., Borisjuk L.G., Alam M.Z., Raskin I. (2000). Production of Recombinant Proteins in Tobacco Guttation Fluid. Plant Physiol..

[30] https://www.oryzogen.net.

[31] https://www.sigmaaldrich.com.

[32] https://www.thermofisher.com.

[33] https://www.orfgenetics.com.

[34] Ma J.K.-C., Drossard J., Lewis D., Altmann F., Boyle J., Christou P., Cole T., Dale P., van Dolleweerd C.J., Isitt V. (2015). Regulatory approval and a first-in-human phase I clinical trial of a monoclonal antibody produced in transgenic tobacco plants. Plant Biotechnol. J..

[35] Hernández-Velázquez A., López-Quesada A., Ceballo-Cámara Y., Cabrera-Herrera G., Tiel-González K., Mirabal-Ortega L., Pérez-Martínez M., Pérez-Castillo R., Rosabal-Ayán Y., Ramos-González O. (2015). Tobacco seeds as efficient production platform for a biologically active anti-HBsAg monoclonal antibody. Transgenic Res..

[36] Sabalza M., Madeira L., van Dolleweerd C., Ma J.K., Capell T., Christou P. (2012). Functional characterization of the recombinant HIV-neutralizing monoclonal antibody 2F5 produced in maize seeds. Plant Mol. Biol..

[37] Paul M.J., Teh A.Y., Twyman R.M., Ma J.K. (2013). Target product selection—Where can molecular pharming make the difference?. Curr. Pharm..

[38] Biesgen C., Hillebrand H., Herbers K. (2002). Technical enzymes produced in transgenic plants. Phytochem. Rev..

[39] Hood E., Witcher D., Maddock S., Meyer T., Baszczynski C., Bailey M., Flynn P., Register J., Marshall L., Bond D. (1997). Commercial production of avidin from transgenic maize: Characterization of transformant, production, processing, extraction and purification. Mol. Breed..

[40] Krishnan A., Woodard S., Howard J., Hood E. (2014). TryZean™: An animal-free alternative to bovine trypsin. Commercial Plant-Produced Recombinant Protein Products. Biotechnology in Agriculture and Forestry.

[41] Bornke F., Broer I. (2010). Tailoring plant metabolism for the production of novel polymers and platform chemicals. Curr. Opin. Plant Biol..

[42] Xu X., Zhang Y., Meng Q., Meng K., Zhang W., Zhou X., Luo H., Chen R., Yang P., Yao B. (2013). Overexpression of a fungal beta-mannanase from *Bispora* sp. MEY-1 in maize seeds and enzyme characterization. PLoS ONE.

[43] http://www.sigmaaldrich.com.

[44] http://www.agrenvec.com.

[45] http://www.orfgenetics.com.

[46] http://www.sifcosmetics.com.

[47] http://www.invitria.com.

[48] http://www.kbpllc.com.

[49] http://www.collplant.com.

[50] http://www.nbms.co.kr.

[51] http://gndp.cigb.edu.cu.

[52] http://www.syngenta.com.

[53] http://www.originseed.com.cn.

[54] http://www.nexgen.com.

[55] Wu T., Kerbler S.M., Fernie A.R., Zhang Y. (2021). Plant cell cultures as heterologous bio-factories for secondary metabolite production. Plant Commun..

[56] Schillberg S., Raven N., Fischer R., Twyman R., Schiermeyer A. (2013). Molecular farming of pharmaceutical proteins using plant suspension cell and tissue cultures. Curr. Pharm. Des..

[57] Schiermeyer A. (2020). Optimizing product quality in molecular farming. Curr. Opin. Biotechnol..

[58] Dicker M., Tschofen M., Maresch D., König J., Juarez P., Orzaez D., Altmann F., Steinkellner H., Strasser R. (2016). Transient Glyco-Engineering to Produce Recombinant IgA1 with Defined N- and O-Glycans in Plants. Front. Plant Sci..

[59] Mercx S., Smargiasso N., Chaumont F., De Pauw E., Boutry M., Navarre C. (2017). Inactivation of the β(1,2)xylosyl transferase and the α(1,3)fucosyltransferase genes in *Nicotiana tabacum* BY2 cells by a multiplex CRISPR/Cas9 strategy results in glycoproteins without plant specific glycans. Front. Plant Sci..

[60] Hanania U., Ariel T., Tekoah Y., Fux L., Gubbay Y., Weiss M., Oz D., Azulay Y., Turbovsky A., Forster Y. (2017). Establishment of a tobacco BY2 cell line devoid of plant specific xylose and fucose as a platform for the production of biotherapeutic proteins. Plant Biotechnol. J..

[61] Shaaltiel Y., Hashmueli S., Bartfeld D., Baum G., Ratz T., Mizrachi E., Forster Y. (2012). System and Method for Production of Antibodies in Plant Cell Culture. Patent.

[62] Moon K.B., Park J.S., Park Y.I., Song I.J., Lee H.J., Cho H.S., Jeon J.H., Kim H.S. (2019). Development of Systems for the Production of Plant-Derived Biopharmaceuticals. Plants.

[63] Kwon J.Y., Yang Y.S., Cheon S.H., Nam H.J., Jin G.H., Kim D.I. (2013). Bioreactor engineering using disposable technology for enhanced production of hCTLA4Ig in transgenic rice cell cultures. Biotechnol. Bioeng..

[64] Huang L.-F., Tan C.-C., Yeh J.-F., Liu H.-Y., Liu Y.-K., Ho S.-L., Lu C.-A. (2015). Efficient secretion of recombinant proteins from rice suspension-cultured cells modulated by the choice of signal peptide. PLoS ONE.

[65] Jung J.-W., Kim N.-S. (2019). Production of functional recombinant cyclic citrullinated peptide monoclonal antibody in transgenic rice cell suspension culture. Transgenic. Res..

[66] Islam M.R., Kim N.S., Jung J.W., Kim H.B., Han S.C., Yang M.S. (2018). Spontaneous pepsin C-catalyzed activation of human pepsinogen C in transgenic rice cell suspension culture: Production and characterization of human pepsin C. Enzym. Microb. Technol..

[67] Nam H.J., Kwon J.Y., Choi H.Y., Kang S.H., Jung H.S., Kim D.I. (2017). Production and Purification of Recombinant Glucocerebrosidase in Transgenic Rice Cell Suspension Cultures. Appl. Biochem. Biotechnol..

[68] Lu C.-A., Lim E.-K., Yu S.-M. (1998). Sugar response sequence in the promoter of a rice α-amylase gene serves as a transcriptional enhancer. J. Biol. Chem..

[69] Chung N.D., Kim N.S., Giap D.V., Jang S.H., Oh S.M., Jang S.H., Kim T.G., Jang Y.S., Yang M.S. (2014). Production of functional human vascular endothelial growth factor(165) in transgenic rice cell suspension cultures. Enzyme Microb. Technol..

[70] Tekoah Y., Shulman A., Kizhner T., Ruderfer I., Fux L., Nataf Y., Bartfeld D., Ariel T., Gingis–Velitski S., Hanania U. (2015). Large-scale production of pharmaceutical proteins in plant cell culture—The protalix experience. Plant Biotechnol. J..

[71] Rosales-Mendoza S., Tello-Olea M.A. (2015). Carrot Cells: A Pioneering Platform for Biopharmaceuticals Production. Mol. Biotechnol..

[72] Hellwig S., Drossard J., Twyman R.M., Fischer R. (2004). Plant cell cultures for the production of recombinant proteins. Nat. Biotechnol..

[73] Santos R.B., Chandrasekar B., Mandal M.K., Kaschani F., Kaiser M., Both L., van der Hoorn R.A.L., Schiermeyer A., Abranches R. (2018). Low protease content in *Medicago truncatula* cell cultures facilitates recombinant protein production. Biotechnol. J..

[74] Huang T.-K., Falk B.W., Dandekar A.M., McDonald K.A. (2018). Enhancement of Recombinant Protein Production in Transgenic *Nicotiana benthamiana* Plant Cell Suspension Cultures with Co-Cultivation of *Agrobacterium* Containing Silencing Suppressors. Int. J. Mol. Sci..

[75] Sukenik S.C., Karuppanan K., Li Q., Lebrilla C.B., Nandi S., McDonald K.A. (2018). Transient Recombinant Protein Production in Glycoengineered Nicotiana benthamiana Cell Suspension Culture. Int. J. Mol. Sci..

[76] Donini M., Marusic C. (2019). Current state-of-the-art in plant-based antibody production systems. Biotechnol. Lett..

[77] Chen Q., Lai H. (2015). Gene delivery into plant cells for recombinant protein production. Biomed. Res. Int..

[78] Tyurin A.A., Suhorukova A.V., Kabardaeva K.V., Goldenkova-Pavlova I.V. (2020). Transient Gene Expression is an Effective Experimental Tool for the Research into the Fine Mechanisms of Plant Gene Function: Advantages, Limitations, and Solutions. Plants.

[79] Norkunas K., Harding R., Dale J., Dugdale B. (2018). Improving agroinfiltration-based transient gene expression in Nicotiana benthamiana. Plant Methods.

[80] Yamamoto T., Hoshikawa K., Ezura K., Okazawa R., Fujita S., Takaoka M., Mason H.S., Ezura H., Miura K. (2018). Improvement of the transient expression system for production of recombinant proteins in plants. Sci. Rep..

[81] Vaquero C., Sack M., Chandler M.J., Drossard J., Schuster F., Monecke M., Schillberg S., Fischer R. (1999). Transient expression of a tumor-specific single-chain fragmentand a chimeric antibody in tobacco leaves. Proc. Natl. Acad. Sci. USA.

[82] D’Aoust M.A., Couture M.M., Charland N., Trépanier S., Landry N., Ors F., Vézina L.P. (2010). The production of hemagglutinin based virus-like particles in plants: A rapid, efficient and safe response to pandemic influenza. Plant Biotechnol. J..

[83] Shoji Y., Chichester J.A., Bi H., Musiychuk K., de la Rosa P., Goldschmidt L., Horsey A., Ugulava N., Palmer G.A., Mett V. (2008). Plant-expressed HA as a seasonal influenza vaccine candidate. Vaccine.

[84] Shoji Y., Chichester J.A., Jones M., Manceva S.D., Damon E., Mett V., Musiychuk K., Bi H., Farrance C., Shamloul M. (2011). Plant-based rapid production of recombinant subunit hemagglutinin vaccines targeting H1N1 and H5N1 influenza. Hum. Vaccin..

[85] Na W., Park N., Yeom M., Song D. (2015). Ebola outbreak in Western Africa 2014: What is going on with Ebola virus?. Clin. Exp. Vaccine Res..

[86] Rybicki E.P. (2020). Plant molecular farming of virus-like nanoparticles as vaccines and reagents. Wiley Interdiscip. Rev. Nanomed. Nanobiotechnol..

[87] https://medicago.com/en/our-science/our-vaccine-candidates/novovirus/.

[88] Pillet S., Couillard J., Trépanier S., Poulin J.-F., Yassine-Diab B., Guy B., Ward B.J., Landry N. (2019). Immunogenicity and safety of a quadrivalent plant-derived virus like particle influenza vaccine candidate—Two randomized Phase II clinical trials in 18 to 49 and ≥50 years old adults. PLoS ONE.

[89] Capell T., Twyman R.M., Armario-Najera V., Ma J.K., Schillberg S., Christou P. (2020). Potential Applications of Plant Biotechnology against SARS-CoV-2. Trends Plant Sci..

[90] Rosales-Mendoza S., Comas-García M., Korban S.S. (2020). Challenges and Opportunities for the Biotechnology Research Community during the Coronavirus Pandemic. Trends Biotechnol..

[91] Hemmati F., Hemmati-Dinarvand M., Karimzade M., Rutkowska D., Eskandari M.H., Khanizadeh S., Afsharifar A. (2022). Plant-Derived VLP: A Worthy Platform to Produce Vaccine against SARS-CoV-2. Biotechnol. Lett..

[92] Maharjan P.M., Choe S. (2021). Plant-Based COVID-19 Vaccines: Current Status, Design, and Development Strategies of Candidate Vaccines. Vaccines.

[93] Diego-Martin B., González B., Vazquez-Vilar M., Selma S., Mateos-Fernández R., Gianoglio S., Fernández-del-Carmen A., Orzáez D. (2020). Pilot Production of SARS-CoV-2 Related Proteins in Plants: A Proof of Concept for Rapid Repurposing of Indoor Farms into Biomanufacturing Facilities. Front. Plant Sci..

[94] Kumar M., Kumari N., Thakur N., Bhatia S.K., Saratale G.D., Ghodake G., Mistry B.M., Alavilli H., Kishor D.S., Du X. (2021). A Comprehensive Overview on the Production of Vaccines in Plant-Based Expression Systems and the Scope of Plant Biotechnology to Combat against SARS-CoV-2 Virus Pandemics. Plants.

[95] Peyret H., Steele J.F.C., Jung J.-W., Thuenemann E.C., Meshcheriakova Y., Lomonossoff G.P. (2021). Producing Vaccines against Enveloped Viruses in Plants: Making the Impossible, Difficult. Vaccines.

[96] Mamedov T., Yuksel D., Ilgın M., Gürbüzaslan I., Gulec B., Mammadova G., Ozdarendeli A., Yetiskin H., Kaplan B., Islam Pavel S.T. (2021). Production and Characterization of Nucleocapsid and RBD Cocktail Antigens of SARS-CoV-2 in Nicotiana Benthamiana Plant as a Vaccine Candidate against COVID-19. Vaccines.

[97] Rybicki E.P. (2014). Plant-based vaccines against viruses. Virol. J..

[98] Clarke J.L., Paruch L., Dobrica M.-O., Caras I., Tucureanu C., Onu A., Ciulean S., Stavaru C., Eerde A., Wang Y. (2017). Lettuce-produced hepatitis C virus E1E2 heterodimer triggers immune responses in mice and antibody production after oral vaccination. Plant Biotech. J..

[99] Naupu P.N., van Zyl A.R., Rybicki E.P., Hitzeroth I.I. (2020). Immunogenicity of Plant-Produced Human Papillomavirus (HPV) Virus-Like Particles (VLPs). Vaccine.

[100] Kasinger S.L.E., Dent M.W., Mahajan G., Hamorsky K.T., Matoba N. (2019). A novel anti-HIV-1 bispecific bNAb-lectin fusion protein engineered in a plant-based transient expression system. Plant Biotechnol. J..

[101] Nessa M.U., Rahman M.A., Kabir Y. (2020). Plant-Produced Monoclonal Antibody as Immunotherapy for Cancer. Biomed. Res. Int..

[102] Buyel J.F. (2018). Plants as sources of natural and recombinant anti-cancer agents. Biotechnol. Adv..

[103] Gengenbach B.B., Keil L.L., Opdensteinen P., Müschen C.R., Melmer G., Lentzen H., Bührmann J., Buyel J.F. (2019). Comparison of microbial and transient expression (tobacco plants and plant-cell packs) for the production and purification of the anticancer mistletoe lectin viscumin. Biotechnol. Bioeng..

[104] Knodler M.J., Buyel F. (2021). Plant-made immunotoxin building blocks: A roadmap for producing therapeutic antibody-toxin fusions. Biotechnol. Adv..

[105] Nirenberg M.W., Matthaei H.J. (1961). The Dependence of Cell-Free Protein Synthesis In *E. coli* Upon Naturally Occurring or Synthetic Polyribonucleotides. Proc. Natl. Acad. Sci. USA.

[106] Buntru M., Vogel S., Finnern R., Schillberg S., Schillberg S., Spiegel H. (2022). Plant-Based Cell-Free Transcription and Translation of Recombinant Proteins. Recombinant Proteins in Plants. Methods in Molecular Biology.

[107] Schillberg S., Raven N., Spiegel H., Rasche S., Buntru M. (2019). Critical analysis of the commercial potential of plants for the production of recombinant proteins. Front. Plant. Sci..

[108] Buntru M., Vogel S., Spiegel H., Schillberg S. (2014). Tobacco BY-2 cell-free lysate: An alternative and highly-productive plant-based in vitro translation system. BMC Biotechnol..

[109] Buntru M., Vogel S., Stoff K., Spiegel H., Schillberg S. (2015). A versatile coupled cell-free transcription-translation system based on tobacco BY-2 cell lysates. Biotechnol. Bioeng..

[110] www.leniobio.com/alice.

[111] Havenith H., Kern C., Rautenberger P., Spiegel H., Szardenings M., Ueberham E., Lehmann J., Buntru M., Vogel S., Treudler R. (2017). Combination of two epitope identification techniques enables the rational design of soy allergen Gly m 4 mutants. Biotechnol. J..

[112] Huck N.V., Leissing F., Majovsky P., Buntru M., Aretz C., Flecken M., Müller J.P., Vogel S., Schillberg S., Hoehenwarter W. (2017). Combined 15N-labeling and tandemMOAC quantifies phosphorylation of MAP kinase substrates downstream of MKK7 in Arabidopsis. Front. Plant Sci..

[113] Spiegel H., Stöger E., Twyman R.M., Buyel J.F., Allison R., Kermode L.J. (2018). Current Status and Perspectives of the Molecular Farming Landscape. Molecular Pharming: Applications, Challenges, and Emerging Areas.

[114] Shanmugaraj B., Bulaon C.J.I., Malla A., Phoolcharoen W. (2021). Biotechnological insights on the expression and production of antimicrobial peptides in plants. Molecules.

[115] Fischer R., Schillberg S., Buyel F.J., Twyman M.R. (2013). Commercial Aspects of Pharmaceutical Protein Production in Plants. Curr. Pharm. Des..

[116] Sethi L., Kumari K., Dey N. (2021). Engineering of Plants for Efficient Production of Therapeutics. Mol. Biotechnol..

[117] Rozov S.M., Deineko E.V. (2019). Strategies for Optimizing Recombinant Protein Synthesis in Plant Cells: Classical Approaches and New Directions. Mol. Biol..

[118] Dong O.X., Ronald P.C. (2021). Targeted DNA insertion in plants. Proc. Natl. Acad. Sci. USA.

[119] Iwase A., Ishii H., Aoyagi H., Ohme-Takagi M., Tanaka H. (2005). Comparative analyses of the gene expression profiles of Arabidopsis intact plant and cultured cells. Biotechnol. Lett..

[120] Tanurdzic M., Vaughn M.W., Jiang H., Lee T.J., Slotkin R.K., Sosinski B., Thompson W.F., Doerge R.W., Martienssen R.A. (2008). Epigenomic consequences of immortalized plant cell suspension culture. PLoS Biol..

[121] Rozov S.M., Permyakova N.V., Sidorchuk Y.V., Deineko E.V. (2022). Optimization of Genome Knock-In Method: Search for the Most Efficient Genome Regions for Transgene Expression in Plants. Int. J. Mol. Sci..

[122] Permyakova N.V., Marenkova T.V., Belavin P.A., Zagorskaya A.A., Sidorchuk Y.V., Uvarova E.A., Kuznetsov V.V., Rozov S.M., Deineko E.V. (2021). Assessment of the Level of Accumulation of the dIFN Protein Integrated by the Knock-In Method into the Region of the Histone H3.3 Gene of *Arabidopsis thaliana*. Cells.

[123] Kim T.G., Lee H.J., Jang Y.S., Shin Y.J., Kwon T.H., Yang M.S. (2008). Co-expression of proteinase inhibitor enhances recombinant human granulocyte-macrophage colony stimulating factor production in transgenic rice cell suspension culture. Protein. Expr. Purif..

[124] Niemer M., Mehofer U., Torres Acosta J.A., Verdianz M., Henkel T., Loos A., Strasser R., Maresch D., Rademacher T., Steinkellner H. (2014). The human anti-HIVantibodies 2F5, 2G12, and PG9 differint heir susceptibility to proteolytic degradation: Down-regulation of endogenous serine and cysteine proteinase activities could improve antibody production in plant-based expression platforms. Biotechnol. J..

[125] Kim N.S., Kim T.G., Kim O.H., Ko E.M., Jang Y.S., Jung E.S., Kwon T.H., Yang M.S. (2008). Improvement of recombinant hGM-CSF production by suppression of cysteine proteinase gene expression using RNA interference in a transgenic rice culture. Plant Mol. Biol..

[126] Xu J., Ge X., Dolan M.C. (2011). Towards high-yield production of pharmaceutical proteins with plant cell suspension cultures. Biotechnol. Adv..

[127] Xu J., Dolan M.C., Medrano G., Cramer C.L., Weathers P.J. (2012). Green factory: Plants as bioproduction platforms for recombinant proteins. Biotechnol. Adv..

[128] Rozov S.M., Permyakova N.V., Deineko E.V. (2018). Main Strategies of Plant Expression System Glycoengineering for Producing Humanized Recombinant Pharmaceutical Proteins. Biochemistry.

[129] Pogson B.J., Ganguly D., Albrecht-Borth V. (2015). Insights into chloroplast biogenesis and development. Biochim. Biophys. Acta.

[130] Mellor S.B., Behrendorff J.B.Y.H., Nielsen A.Z., Jensen P.E., Pribil M. (2018). Non-photosynthetic plastids as hosts for metabolic engineering. Essays Biochem..

[131] Farquhar J., Zerkle A.L., Bekker A. (2011). Geological constraints on the origin of oxygenic photosynthesis. Photosynth. Res..

[132] Taylor N.L., Stroher E., Millar A.H. (2014). Arabidopsis organelle isolation and characterization. Methods Mol. Biol..

[133] Palmer J.D., Osorio B., Aldrich J., Thompson W.F. (1987). Chloroplast DNA evolution among legumes: Loss of a large inverted repeat occurred prior to other sequence rearrangements. Curr. Genet..

[134] Daniell H., Lin C.-S., Yu M., Chang W.-J. (2016). Chloroplast genomes: Diversity, evolution, and applications in genetic engineering. Genome Biol..

[135] Barkan A. (2011). Expression of plastid genes: Organelle-specific elaborations on a prokaryotic scaffold. Plant Physiol..

[136] Prikryl J., Rojas M., Schuster G., Barkan A. (2011). Mechanism of RNA stabilization and translational activation by a pentatricopeptide repeat protein. Proc. Natl. Acad. Sci. USA.

[137] Gawronski P., Jensen P.E., Karpinski S., Leister D., Scharff L.B. (2018). Pausing of chloroplast ribosomes is induced by multiple features and is linked to the assembly of photosynthetic complexes. Plant Physiol..

[138] Vries J., Archibald J.M. (2018). Plastid genomes. Curr. Biol..

[139] Oldenburg D.J., Bendich A.J. (2015). DNA maintenance in plastids and mitochondria of plants. Front. Plant Sci..

[140] Mehmood F., Abdullah U.Z., Shahzadi I., Ahmed I., Waheed M.T., Poczai P., Mirza B. (2020). Plastid genomics of Nicotiana (Solanaceae): Insights into molecular evolution, positive selection and the origin of the maternal genome of Aztec tobacco (Nicotiana rustica). PeerJ..

[141] Sakamoto W., Takami T. (2018). Chloroplast DNA dynamics: Copy number, quality control and degradation. Plant Cell Physiol..

[142] Rose R.J. (2019). Sustaining Life: Maintaining Chloroplasts and Mitochondria and their Genomes in Plants. Yale J. Biol. Med..

[143] Zoschke R., Bock R. (2018). Chloroplast Translation: Structural and Functional Organization, Operational Control, and Regulation. Plant Cell.

[144] Tadini L., Jeran N., Peracchio C., Masiero S., Colombo M., Pesaresi P. (2020). The plastid transcription machinery and its coordination with the expression of nuclear genome: Plastid-Encoded Polymerase, Nuclear-Encoded Polymerase and the Genomes Uncoupled 1-mediated retrograde communication. Phil. Trans. R Soc..

[145] Jarvis P., Lopez-Juez E. (2013). Biogenesis and homeostasis of chloroplasts and other plastids. Nat. Rev. Mol. Cell Biol..

[146] Bock R. (2015). Engineering plastid genomes: Methods, tools, and applications in basic research and biotechnology. Annu. Rev. Plant Biol..

[147] McBride K.E., Svab Z., Schaaf D.J., Hogan P.S., Stalker D.M., Maliga P. (1995). Amplification of a chimeric Bacillus gene in chloroplasts leads to an extraordinary level of an insecticidal protein in tobacco. Biotechnology.

[148] Fuentes P., Armarego-Marriott T., Bock R. (2018). Plastid transformation and its application in metabolic engineering. Curr. Opin. Biotechnol..

[149] Ahmad N., Mehmood M.A., Malik S. Recombinant protein production in microalgae: Emerging trends. Protein Pept. Lett..

[150] Dreesen I.A.J., Charpin-El Hamri G., Fussenegger M. (2010). Heat-stable oral alga-based vaccine protects mice from *Staphylococcus aureus* infection. J. Biotechnol..

[151] Gregory J.A., Topol A.B., Doerner D.Z., Mayfield S. (2013). Alga-produced cholera toxin-Pfs25 fusion proteins as oral vaccines. Appl. Environ. Microbiol..

[152] Boynton J.E., Gillham N.W., Harris E.H., Hosler J.P., Johnson A.M., Jones A.R., Randolf-Anderson B.L., Robertson D., Klein T.M., Shark K.B. (1988). Chloroplast transformation in *Chlamydomonas* with high velocity microprojectiles. Science.

[153] Svab Z., Hajdukiewicz P., Maliga P. (1990). Stable transformation of plastids in higher plants. Proc. Natl. Acad. Sci. USA.

[154] Tamburino R., Marcolongo L., Sannino L., Ionata E., Scotti N. (2022). Plastid Transformation: New Challenges in the Circular Economy Era. Int. J. Mol. Sci..

[155] Jin S., Daniell H. (2015). The engineered chloroplast genome just got smarter. Trends in Plant Sci..

[156] Ruf S., Forner J., Hasse C., Kroop X., Seeger S., Schollbach L., Schadach A., Bock R. (2019). High-efficiency generation of fertile transplastomic Arabidopsis plants. Nat. Plants.

[157] Cutolo E.A., Mandalà G., Dall’Osto L., Bassi R. (2022). Harnessing the Algal Chloroplast for Heterologous Protein Production. Microorganisms.

[158] Kumari U., Singh R., Ray T., Rana S., Saha P., Malhotra K., Daniell H. (2019). Validation of leaf enzymes in the detergent and textile industries: Launching of a new platform technology. Plant Biotechnol. J..

[159] Sánchez E.A.E., Castillo J.A.T., Cruz Q.R., García S.R.S. (2018). Biotechnological Applications of Plastid Foreign Gene Expression. Plant Growth and Regulation-Alterations to Sustain Unfavorable Conditions.

[160] Lu Y., Rijzaani H., Karcher D., Ruf S., Bock R. (2013). Efficient metabolic pathway engineering in transgenic tobacco and tomato plastids with synthetic multigene operons. Proc. Natl. Acad. Sci. USA.

[161] Kwak S.Y., Lew T.T.S., Sweeney C.J., Koman V.B., Wong M.H., Bohmert-Tatarev K., Snell K.D., Seo J.S., Chua N.H., Strano M.S. (2019). Chloroplast-selective gene delivery and expression in planta using chitosan-complexed single-walled carbon nanotube carriers. Nat. Nanotechnol..

[162] Chen Z., Zhao J., Cao J., Zhao Y., Huang J., Zheng Z., Li W., Jang S., Qiao J., Xing B. (2022). Opportunities for graphene, single-walled and multi-walled carbon nanotube applications in agriculture: A review. Crop. Des..

[163] Thagun C., Chuah J.A., Numata K. (2019). Targeted gene delivery into various plastids mediated by clustered cell-penetrating and chloroplast-targeting peptides. Adv. Sci..

[164] Hanson M.R., Gray B.N., Ahner B.A. (2013). Chloroplast transformation for engineering of photosynthesis. J. Exp. Bot..

[165] Dauvillee D., Hilbig L., Preiss S., Johanningmeier U. (2004). Minimal extent of sequence homology required for homologous recombination at the psbA locus in *Chlamydomonas reinhardtii* chloroplasts using PCR-generated DNA fragments. Photosynth. Res..

[166] Saski C., Lee S.B., Fjellheim S., Guda C., Jansen R.K., Luo H., Tomkins J., Rognli O.A., Daniell H., Clarke J.L. (2007). Complete chloroplast genome sequences of Hordeum vulgare, Sorghum bicolor and Agrostis stolonifera, and comparative analyses with other grass genomes. Appl. Genet..

[167] Yarra R. (2020). Plastome engineering in vegetable crops: Current status and future prospects. Mol. Biol. Rep..

[168] Khan M.S., Maliga P. (1999). Fluorescent antibiotic resistance marker for tracking plastid transformation in higher plants. Nat. Biotechnol.

[169] Yoo B.C., Yadav N.S., Orozco E.M., Sakai H. (2020). Cas9/gRNA-mediated genome editing of yeast mitochondria and Chlamydomonas chloroplasts. PeerJ.

[170] Tang N., Xia Y., Zhan Y., Dan J., Yu M., Bu X., Cao M. (2020). Improvement of Chloroplast Transformation Using CRISPR/Cas9. J. Biobased Mater. Bioenergy.

[171] Liere K., Börner T. (2007). Transcription and transcriptional regulation in plastids. Top. Curr. Genet..

[172] Hajdukiewicz P.T.J., Allison L.A., Maliga P. (1997). The two RNA polymerases encoded by the nuclear and the plastid compartments transcribe distinct groups of genes in tobacco plastids. EMBO J..

[173] Bock R. (2014). Engineering chloroplasts for high-level foreign protein expression. Chloroplast. Biotechnol..

[174] Ruhlman T., Verma D., Samson N., Daniell H. (2010). The role of heterologous chloroplast sequence elements in transgene integration and expression. Plant Physiol..

[175] Bohne A.-V., Ruf S., Börner T., Bock R. (2007). Faithful transcription initiation from a mitochondrial promoter in transgenic plastids. Nucleic Acids Res..

[176] Eberhard S., Drapier D., Wollman F.-A. (2002). Searching limiting steps in the expression of chloroplast-encoded proteins: Relations between gene copy number, transcription, transcript abundance and translation rate in the chloroplast of Chlamydomonas reinhardtii. Plant J..

[177] Herz S., Füßl M., Steiger S., Koop H.-U. (2005). Development of novel types of plastid transformation vectors and evaluation of factors controlling expression. Transgenic. Res..

[178] Oey M., Lohse M., Kreikemeyer B., Bock R. (2009). Exhaustion of the chloroplast protein synthesis capacity by massive expression of a highly stable protein antibiotic. Plant J..

[179] Zhou F., Badillo-Corona J.A., Karcher D., Gonzalez-Rabade N., Piepenburg K., Borchers A.-M.I., Maloney A.P., Kavanagh T.A., Gray J.C., Bock R. (2008). High-level expression of HIV antigens from the tobacco and tomato plastid genomes. Plant Biotechnol. J..

[180] Klein R.R., Mullet J.E. (1987). Control of gene expression during higher plant chloroplast biogenesis. Protein synthesis and transcript levels of psbA, psaA-psaB, and rbcL in dark-grown and illuminated barley seedlings. J. Biol. Chem..

[181] Mardanov A.V., Ravin N.V., Kuznetsov B.B., Samigullin T.H., Antonov A.S., Kolganova T.V., Skryabin K.G. (2008). Complete sequence of the duckweed (Lemna minor) chloroplast genome: Structural organization and phylogenetic relationships to other angiosperms. J. Mol. Evol..

[182] Lin C.S., Chen J.J., Huang Y.T., Chan M.T., Daniell H., Chang W.J., Hsu C.-T., Liao D.-C., Wu F.-H., Lin S.-Y. (2015). The location and translocation of ndh genes of chloroplast origin in the Orchidaceae family. Sci. Rep..

[183] Wu J., Liu B., Cheng F., Ramchiary N., Choi S.R., Lim Y.P., Wang X.-W. (2012). Sequencing of chloroplast genome using whole cellular DNA and Solexa sequencing technology. Front. Plant. Sci..

[184] Yu Y., Yu P.C., Chang W.J., Yu K., Lin C.S. (2020). Plastid transformation: How does it work? Can it be applied to crops? What can it offer?. Int. J. Mol. Sci..

[185] Golczyk H., Greiner S., Wanner G., Weihe A., Bock R., Börner T., Herrmann R.G. (2014). Chloroplast DNA in mature and senescing leaves: A reappraisal. Plant Cell.

[186] Greiner S., Golczyk H., Malinova I., Pellizzer T., Bock R., Börner T., Herrmann R.G. (2020). Chloroplast nucleoids are highly dynamic in ploidy, number, and structure during angiosperm leaf development. Plant J..

[187] Teske D., Peters A., Moöllers A., Fischer M. (2020). Genomic profiling: The strengths and limitations of chloroplast genome-based plant variety authentication. J. Agric. Food Chem..

[188] Tetsuaki Osafune S.S., Hase E. (2012). Proplastids of dark-grown wax-rich cells of *Euglena gracilis*. Regul. Chloroplast Biog..

[189] Maliga P. (2022). Engineering the plastid and mitochondrial genomes of flowering plants. Nat. Plants.

[190] Devarumath R.M., Nerkar G.A., Farsangi F.J., Nikam A.A., Babu K.H., Tiwari A.K., Singh A.K., Lal M. (2015). Embracing biotechnology methods for crop improvement research in sugarcane. Current Status of Sugarcane Research in India.

[191] Mustafa G., Khan M.S. (2021). Transmission of Engineered Plastids in Sugarcane, a C4 Monocotyledonous Plant, Reveals that Sorting of Preprogrammed Progenitor Cells Produce Heteroplasmy. Plants.

[192] Maliga P., Bock R., Knoop V. (2012). Plastid transformation in flowering plants. Genomomics of Chloroplasts Mitochondria.

[193] Kumar S., Dhingra A., Daniel H. (2004). Stable transformation of the cotton plastid genome and maternal inheritance of transgenes. Plant Mol. Biol..

[194] Huang F.C., Klaus S.M.J., Herz S., Zou Z., Koop H.U., Golds T.J. (2002). Efficient plastid transformation in tobacco using the aphA-6 gene and kanamycin selection. Mol. Genet. Genom..

[195] Day A., Goldschmidt-Clermont M. (2011). The chloroplast transformation toolbox: Selectable markers and marker removal. Plant Biotechnol. J..

[196] Bock R. (2014). Genetic engineering of the chloroplast: Novel tools and new applications. Curr. Opin. Biotechnol..

[197] Borovinskaya M.A., Shoji S., Holton J.M., Fredric K., Cate J.H.D. (2007). A steric block in translation caused by the antibiotic spectinomycin. ACS Chem. Biol..

[198] Li W., Ruf S., Bock R. (2011). Chloramphenicol acetyltransferase as selectable marker for plastid transformation. Plant Mol. Biol..

[199] Barone P., Zhang X.H., Widholm J.M. (2009). Tobacco plastid transformation using the feedback-insensitive anthranilate synthase [alpha]-subunit of tobacco (ASA2) as a new selectable marker. J. Exp. Bot..

[200] Verma D., Daniell H. (2007). Chloroplast vector systems for biotechnology applications. Plant Physiol..

[201] Khakhlova O., Bock R. (2006). Elimination of deleterious mutations in plastid genomes by gene conversion. Plant J..

[202] Ye G.N., Colburn S., Xu C.W., Hajdukiewicz P.T.J., Staub J.M. (2003). Persistence of unselected transgenic DNA during a plastid transformation and segregation approach to herbicide resistance. Plant Physiol..

[203] Shimizu M., Goto M., Hanai M., Shimizu T., Izawa N., Kanamoto H., Tomizawa K., Yokota A., Kobayashi H. (2008). Selectable tolerance to herbicides by mutated acetolactate synthase genes integrated into the chloroplast genome of tobacco. Plant Physiol..

[204] Dufourmantel N., Dubald M., Matringe M., Canard H., Garcon F., Job C., Kay E., Wisniewski J.P., Ferullo J.M., Pelissier B. (2007). Generation and characterization of soybean and marker-free tobacco plastid transformants over-expressing a bacterial 4-hydroxyphenylpyruvate dioxygenase which provides strong herbicide tolerance. Plant Biotechnol. J..

[205] Okuzaki A., Tsuda M., Konagaya K.I., Tabei Y. (2020). A novel strategy for promoting homoplasmic plastid transformant production using the barnase–barstar system. Plant Biotechnol..

[206] Hartley R.W. (1988). Barnase and barstar: Expression of its cloned inhibitor permits expression of a cloned ribonuclease. J. Mol. Biol..

[207] Abe K., Oshima M., Akasaka M., Konagaya K., Nanasato Y., Okuzaki A., Taniguchi Y., Tanaka J., Tabei Y. (2018). Development and characterization of transgenic dominant male sterile rice toward an outcross-based breeding system. Breed. Sci..

[208] Dyo Y.M., Purton S. (2018). The algal chloroplast as a synthetic biology platform for production of therapeutic proteins. Microbiology.

[209] Zhou F., Karcher D., Bock R. (2007). Identification of a plastid Intercistronic Expression Element (IEE) facilitating the expression of stable translatable monocistronic mRNAs from operons. Plant J..

[210] Liu Y., Medina R., Goffinet B. (2014). 350 my of mitochondrial genome stasis in mosses, an early land plant lineage. Mol. Biol. Evol..

[211] Sloan D.B., Alverson A.J., Chuckalovcak J.P., Wu M., McCauley D.E., Palmer J.D., Taylor D.R. (2012). Rapid evolution of enormous, multichromosomal genomes in flowering plant mitochondria with exceptionally high mutation rates. PLoS Biol..

[212] Wu Z.Q., Liao X.Z., Zhang X.N., Tembrock L.R., Broz A. (2022). Genomic architectural variation of plant mitochondria—A review of multichromosomal structuring. J. Syst. Evol..

[213] Sloan D.B., Warren J.M., Williams A.M., Wu Z., Abdel-Ghany S.E., Chicco A.J., Havird J.C. (2018). Cytonuclear integration and co-evolution. Nat. Rev. Genet..

[214] Sloan D.B., Wu Z., Sharbrough J. (2018). Correction of persistent errors in Arabidopsis reference mitochondrial genomes. Plant Cell.

[215] Alverson A.J., Rice D.W., Dickinson S., Barry K., Palmer J.D. (2011). Origins and Recombination of the Bacterial-Sized Multichromosomal Mitochondrial Genome of Cucumber. Plant Cell.

[216] Preuten T., Cincu E., Fuchs J., Zoschke R., Liere K., Börner T. (2010). Fewer genes than organelles: Extremely low and variable gene copy numbers in mitochondria of somatic plant cells. Plant J..

[217] Klein M., Eckert-Ossenkopp U., Schmiedeberg I., Brandt P., Unseld M., Brennicke A., Schuster W. (1994). Physical mapping of the mitochondrial genome of Arabidopsis thaliana by cosmid and YAC clones. Plant J..

[218] Backert S., Lurz R., Oyarzabal O.A., Börner T. (1997). High content, size and distribution of single-stranded DNA in the mitochondria of *Chenopodium album* (L.). Plant Mol. Biol..

[219] Gualberto J.M., Kühn K. (2014). DNA-binding proteins in plant mitochondria: Implications for transcription. Mitochondrion.

[220] Weihe A., Daniell H., Chase C. (2004). The Transcription of Plant Organelle Genomes. Molecular Biology and Biotechnology of Plant Organelles: Chloroplasts and Mitochondria.

[221] Bonnefoy N., Remacle C., Fox T.D. (2007). Genetic transformation of *Saccharomyces cerevisiae* and *Chlamydomonas reinhardtii* mitochondria. Methods Cell Biol..

[222] Zhou J., Liu L., Chen J. (2010). Mitochondrial DNA heteroplasmy in *Candida glabrata* after mitochondrial transformation. Eukaryot.

[223] Kazama T., Okuno M., Watari Y., Yanase S., Koizuka C., Tsuruta Y., Susaya H., Toyoda A., Itoh T., Tsutsumi N. (2019). Curing cytoplasmic male sterility via TALEN-mediated mitochondrial genome editing. Nat. Plants.

[224] Arimura S.I., Ayabe H., Sugaya H., Okuno M., Tamura Y., Tsuruta Y., Watari Y., Yanase S., Yamauchi T., Itoh T. (2020). Targeted gene disruption of ATP synthases 6-1 and 6-2 in the mitochondrial genome of *Arabidopsis thaliana* by mitoTALENs. Plant J..

[225] Nakazato I., Okuno M., Zhou C., Itoh T., Tsutsumi N., Takenaka M., Arimura S.I. (2022). Targeted base editing in the mitochondrial genome of Arabidopsis thaliana. Proc. Natl. Acad. Sci. USA.

[226] Koulintchenko M., Konstantinov Y., Dietrich A. (2003). Plant mitochondria actively import DNA via the permeability transition pore complex. EMBO J..

[227] Ibrahim N., Handa H., Cosset A., Koulintchenko M., Konstantinov Y., Lightowlers R.N., Dietrich A., Weber-Lotfi F. (2011). DNA delivery to mitochondria: Sequence specificity and energy enhancement. Pharm. Res..

[228] Tarasenko T.A., Klimenko E.S., Tarasenko V.I., Koulintchenko M.V., Dietrich A., Weber-Lotfi F., Konstantinov Y.M. (2021). Plant mitochondria import DNA via alternative membrane complexes involving various VDAC isoforms. Mitochondrion.

[229] Mileshina D., Koulintchenko M., Konstantinov Y., Dietrich A. (2011). Transfection of plant mitochondria and in organello gene integration. Nucleic Acids Res..

[230] Tarasenko T.A., Tarasenko V.I., Koulintchenko M.V., Klimenko E.S., Konstantinov Y.M. (2019). DNA Import into Plant Mitochondria: Complex approach for in organello and in vivo studies. Biochemistry.

